# Millennial-scale variability of Greenland dust provenance during the last glacial maximum as determined by single particle analysis

**DOI:** 10.1038/s41598-024-52546-x

**Published:** 2024-01-23

**Authors:** Seokhyun Ro, Jonghyeon Park, Hanjin Yoo, Changhee Han, Ahhyung Lee, Yoojin Lee, Minjeong Kim, Yeongcheol Han, Anders Svensson, Jinhwa Shin, Chul-Un Ro, Sungmin Hong

**Affiliations:** 1 Department of Ocean Sciences, Inha University, 100 Inha-ro, Michuhol-gu, Incheon, 22212 Republic of Korea; 2 Division of Glacial Environment Research, Korea Polar Research Institute, 26 Songdomirae-ro, Yeonsu-gu, Incheon, 21990 Republic of Korea; 3 Department of Chemistry, Inha University, 100 Inha-ro, Michuhol-gu, Incheon, 22212 Republic of Korea; 4 Marine Environment Research Department, Ara Consulting and Technology, 30 Songdomirae-ro, Yeonsu-gu, Incheon, 21990 Republic of Korea; 5 Particle Pollution Research and Management Center, Inha University, 36 Gaetbeol-ro, Yeonsu-gu, Incheon, 21999 Republic of Korea; 6 Department of Water Environmental Safety Management, Korea Water Resources Corporation, 200 Sintanjin-ro, Daedeok-gu, Daejeon, 34350 Republic of Korea; 7 Unit of Frontier Exploration, Korea Polar Research Institute, 26 Songdomirae-ro, Yeonsu-gu, Incheon, 21990 Republic of Korea; 8 Centre for Ice and Climate, Niels Bohr Institute, University of Copenhagen, Julian 10 Maries Vej 30, 2100 Copenhagen, Denmark

**Keywords:** Biogeochemistry, Climate sciences, Environmental sciences

## Abstract

Greenland ice core records exhibited 100-fold higher dust concentrations during the Last Glacial Maximum (LGM) than during the Holocene, and dust input temporal variability corresponded to different climate states in the LGM. While East Asian deserts, the Sahara, and European loess have been suggested as the potential source areas (PSAs) for Greenland LGM dust, millennial-scale variability in their relative contributions within the LGM remains poorly constrained. Here, we present the morphological, mineralogical, and geochemical characteristics of insoluble microparticles to constrain the provenance of dust in Greenland NEEM ice core samples covering cold Greenland Stadials (GS)-2.1a to GS-3 (~ 14.7 to 27.1 kyr ago) in the LGM. The analysis was conducted on individual particles in microdroplet samples by scanning electron microscopy with energy dispersive X-ray spectroscopy and Raman microspectroscopy. We found that the kaolinite-to-chlorite (K/C) ratios and chemical index of alteration (CIA) values were substantially higher (K/C: 1.4 ± 0.7, CIA: 74.7 ± 2.9) during GS-2.1a to 2.1c than during GS-3 (K/C: 0.5 ± 0.1, CIA: 65.8 ± 2.8). Our records revealed a significant increase in Saharan dust contributions from GS-2.1a to GS-2.1c and that the Gobi Desert and/or European loess were potential source(s) during GS-3. This conclusion is further supported by distinctly different carbon contents in particles corresponding to GS-2.1 and GS-3. These results are consistent with previous estimates of proportional dust source contributions obtained using a mixing model based on Pb and Sr isotopic compositions in NEEM LGM ice and indicate millennial-scale changes in Greenland dust provenance that are probably linked to large-scale atmospheric circulation variabilities during the LGM.

## Introduction

Airborne mineral dust (hereafter referred to as dust) plays a crucial role in the Earth’s climate system by affecting radiative balance through modifying cloud regimes^1^, disrupting solar and infrared radiation patterns^2^, and altering surface albedo^3^. In addition, dust is an important source of iron, a growth-limiting phytoplankton micronutrient in the open ocean, and therefore, changes the oceanic uptake of atmospheric CO_2_ and global climate^4,5^.

Dust concentrations in central Greenland ice cores show pronounced climate-related variations and a 100-fold increase during the Last Glacial Maximum (LGM, ~ 26–19 ky ago^6^) compared to the Holocene^7^. Higher LGM dust deposition in Greenland was explained by enhanced continental aridity, increased wind strength, and reduced en route wet removal of dust from the atmosphere^8,9^. However, there remains a discrepancy between observed and modeled dust fluxes in Greenland during the LGM possibly caused by source area changes of putative contributors to Greenland dust^9–11^.

Early studies indicated that East Asian deserts were the dominant source of Greenland LGM dust based on observed diverse mineral and isotopic signatures in central Greenland ice^12–15^. However, subsequent studies proposed Saharan dust^16,17^ and European loess deposits^17,18^ made significant contributions. Overall, these studies provided the possibility that dust from multiple potential source areas (PSAs) reached Greenland during the LGM, which emphasized that well-defined geographical dust origins remained elusive. In addition to the LGM dust provenance issue, a millennial-scale record of dust provenance change across the LGM is required, because ice core dust records reveal substantial variations in dust concentrations between the cooler Greenland Stadials (GS)-2.1 and GS-3, reaching its maxima during GS-3 (the so-called GS-3 dust peaks)^8,19^. Knowledge of the relationship between variations in dust provenance and changing dust concentrations across the LGM would help expand our understanding of the climate sensitivity of environmental changes and atmospheric circulation regimes and reduce discrepancies between observed and modeled dust concentrations in Greenland during the LGM.

Despite its importance, existing ice core records offer limited information about how climatic conditions influenced dust provenance variations across the LGM because sampling has inadequately addressed dust provenance changes on millennial timescales. However, in a recent study by Han et al.^16^ that addressed this shortcoming, the Sahara desert was proposed to be an important source, particularly during GS-2.1a to GS-2.1c (~ 16 to 22.6 ky ago) and primary dust provenance was assigned to East Asia during GS-3 (~ 23.4 to 27.5 ky ago). This study combined the compositions of Pb and Sr isotopes as fingerprints to enable the determination of the provenance of dust trapped in Greenland snow and ice^15,16^. However, the broad and overlapping isotopic signatures of PSAs introduce uncertainty to the discrimination of Greenland dust provenance^16^, underscoring the need to validate variations in Greenland LGM dust sources using different analytical methods.

In this study, we investigated the origin of dust particles in 11 discrete ice samples of the North Greenland Eemian Ice Drilling (NEEM) ice core, covering separate periods between GS-2.1a and GS-3 within the LGM, to gain insight into the issues raised above. The analytical method utilized was scanning electron microscopy coupled with energy dispersive X-ray spectrometry (SEM/EDX), which has proven to be an ideal tool for examining the morphology and major elemental compositions of individual particles and determining their mineralogical and geochemical characteristics based on achieved chemical compositions. After identifying the mineralogy of individual particles by SEM/EDX, Raman microspectroscopy (RMS) was conducted on two selected samples to confirm their mineralogies. In order to identify millennial-scale variabilities in major dust sources during the LGM, we then assessed changes in the mineralogical and geochemical characteristics of the samples and investigated potential mechanisms responsible for observed dust provenance changes using a combination of different types of paleo-proxy records.

## Results and discussion

### Particle sizes and morphological properties

Table 1 summarizes the physical and chemical properties of individual particles determined in 11 Greenland LGM ice core samples. Mean diameters of insoluble particles in the samples ranged from 1.6 to 2.6 μm with a mean ± standard deviation (SD) of 2.1 ± 0.3 μm, which were within or slightly greater than mean LGM dust size distributions reported in Greenland ice cores (~ 1.7–2 μm)^8,20^. Small variations in mean particle sizes between samples may be attributed in part to short-term changes in particle size distributions within the LGM, as has been well reported in the dust record of the North Greenland Ice Core Project (NGRIP) deep ice core^8^. Note that no statistical significance at the 95% level of confidence (*p* < 0.05) exists in short-term variations of mean particle size across the LGM (see Supplementary Section S1). All particles in the samples had a diameter of ≤ 5 µm (Table 1), which allows for a more accurate representation of the characteristics of dust particles transported long-range from PSAs than bulk analysis because a few large particles can mask the physiochemical properties of smaller particles.

**Table 1:** Description of the Greenland NEEM ice core samples and summary of particle morphology (size and shape), mineralogical abundances (%), kaolinite-to-chlorite (K/C) ratio, mass concentrations of oxides (wt%), calculated CIA values (mol/mol%), and carbon (C) concentrations (% in atomic fraction) determined using SEM/EDX analysis.

NEEM Sample			N1	N2	N3	N4	N5	N6	N7	N8	N9	N10	N11
Top depth (m)			1490.15	1506.65	1539.65	1550.65	1556.15	1583.65	1589.15	1594.65	1605.65	1616.65	1633.15
Age (yrs b2k)			14,737	15,999	18,498	19,342	19,771	22,082	22,578	23,046	24,145	25,370	27,144
Time period^a^ (yr)			1.66	1.89	1.86	1.90	1.81	2.15	1.95	1.68	2.37	2.48	2.22
Greenland Stadials^b^			GS-2.1a	GS-2.1a	GS-2.1b	GS-2.1b	GS-2.1b	GS-2.1c	GS-2.1c	GS-2.2	GS-3	GS-3	GS-3
Measured particles	Number		191	200	187	196	193	198	193	196	194	198	193
	Diameter (μm)	Mean ± SD	2.1 ± 0.7	2.0 ± 0.7	2.6 ± 0.9	1.8 ± 0.7	2.1 ± 0.8	2.0 ± 0.7	2.2 ± 0.8	2.3 ± 0.8	1.6 ± 0.5	2.3 ± 0.9	2.2 ± 0.8
		Min–Max	1.1–4.7	0.9–4.2	1.1–4.9	0.6–4.9	0.9–4.6	0.9–4.4	0.9–5.0	1.0–5.0	0.9–3.4	0.9–5.0	1.0–4.9
	Aspect ratio	Mean ± SD	1.3 ± 0.2	1.5 ± 0.4	1.5 ± 0.4	1.3 ± 0.2	1.3 ± 0.2	1.5 ± 0.4	1.3 ± 0.2	1.5 ± 0.4	1.4 ± 0.3	1.5 ± 0.4	1.5 ± 0.5
		Min–Max	1.0–2.1	1.0–3.0	1.0–4.1	1.0–2.3	1.0–2.2	1.0–3.2	1.0–2.5	1.0–2.7	1.0–3.0	1.0–3.2	1.0–3.9
Quartz			20.4	18.0	32.6	23.5	22.8	15.7	19.2	24.0	13.9	24.8	21.8
Feldspars^c^			10.5	14.5	17.1	9.7	14.5	12.1	17.1	16.3	9.8	14.1	18.1
Kaolinite			7.3	3.5	2.7	1.5	5.7	4.0	4.7	4.1	1.6	1.5	2.1
Pyrophyllite			–	0.5	0.5	–	–	–	–	–	–	–	–
Chlorite			5.8	2.0	2.7	1.5	2.1	3.5	6.2	4.6	4.6	2.5	3.6
Fe–oxides			0.5	–	0.5	2.6	2.1	–	1.6	1.0	1.6	–	0.5
TiO_2_			0.5	–	0.5	1.0	0.5	–	1.6	–	0.5	–	0.5
Other (alumino)silicates			55.0	61.5	43.3	60.2	52.3	64.7	49.7	50.0	68.0	57.1	53.4
K/C ratio			1.3	1.8	1.0	1.0	2.8	1.1	0.8	0.9	0.3	0.6	0.6
Fe_2_O_3_			7.5	0.6	6.2	9.3	8.2	1.2	7.7	6.8	9.9	1.6	7.2
TiO_2_			0.8	0.2	1.1	0.7	0.3	0.7	0.7	0.4	0.5	0.3	0.4
CaO			1.7	1.8	0.6	2.6	2.4	1.3	2.4	2.1	5.2	3.4	2.6
K_2_O			1.5	1.7	2.6	1.8	1.2	1.8	1.1	2.8	1.2	2.1	2.7
SiO_2_			57.8	60.0	65.1	54.0	57.2	57.6	56.7	58.5	49.6	55.5	57.7
Al_2_O_3_			21.4	23.0	17.8	19.0	21.2	22.2	22.1	19.2	19.7	21.0	20.5
MgO			4.8	5.0	2.6	4.5	4.1	6.1	5.4	4.1	3.4	4.4	4.1
Na_2_O			1.2	1.6	1.1	1.1	1.2	1.0	1.3	1.1	0.6	1.2	1.3
Others			3.4	6.1	2.9	7.0	4.1	8.1	2.5	5.0	10.0	10.4	3.5
CIA			76.4	74.9	75.6	69.4	73.4	78.9	74.2	68.7	62.6	66.9	67.8
C (mean ± SD)			3.4 ± 4.9	8.9 ± 6.9	5.3 ± 4.7	11.4 ± 13.6	5.7 ± 5.9	11.9 ± 8.1	3.2 ± 5.6	6.6 ± 6.9	13.6 ± 13.8	14.1 ± 10.0	6.2 ± 6.1

^a^The time period integrated by each core section^16^.

^b^Sequence of Greenland Stadials (GS) from Rasmussen et al.^19^ and Seierstad et al.^28^.

^c^Plagioclase (Na,Ca–feldspar), K–feldspar, and feldspar mixtures.

Mean aspect ratios (ARs, a ratio of particle length to width) did not vary significantly between samples (Table 1). Mean ARs of ~ 1.5 with small SDs of 0.2–0.5 are close to those (1.3–1.9) observed for Asian, African, and European mineral dust, and did not vary significantly as a function of particle size^21–23^. Our results show that dust morphology in the samples provided no important constraints on the millennial-scale variability of the primary source of Greenland LGM dust (see Supplementary Section S1).

### Validation of mineral identification

Mineral species identified in the samples by SEM/EDX included quartz, feldspars, kaolinite, pyrophyllite, chlorite, mica, mica/chlorite mixed-layer clay (M/C), illite, smectite, illite/smectite mixed-layer clay (I/S), Fe–oxides, and TiO_2_. Previous studies have also reported these minerals in Greenland ice^13,14,17,24,25^.

Further confirmation of mineral types was obtained using Raman spectra for samples nos. N3 and N9, corresponding to GS-2.1b and GS-3, respectively (Table 1). These samples were selected because there were considerable differences in particle size and mineral composition between the two (Table 1). Despite extensive efforts to optimize the RMS measurements of particles contained in ice core samples (see Supplementary Section S2), we encountered Raman spectra exhibiting a strong fluorescence signal followed by the Raman D–G band (hereafter referred to as the F/D–G signal) for 56.6% of particles (Supplementary Fig. S1), which made it difficult to identify mineral species. Interestingly, the proportion of particles exhibiting the F/D–G signal was greater in sample N9 (72.6%) than in N3 (40.6%). The reasons for the compositional differences in minerals exhibiting the F/D–G signal will be discussed in detail in the following section. The presence of particles exhibiting the F/D–G signal emphasizes the need to be careful when using RMS to analyze dust particles in ice cores.

Despite the limitations of using RMS in terms of mineral identification, the presence of particles exhibited the Raman spectra of standard minerals allowed us to determine the presence of different minerals such as quartz, feldspars, chlorite, mica, illite, smectite, pyroxene, amphibole, zeolite, hematite, and rutile (Supplementary Fig. S1). However, some particles did not have Raman features that matched known minerals. Note that the mixed-layer clays (M/C and I/S) could not be identified by their Raman spectra because of the lack of available data in the literature to the best of our knowledge. Individual minerals such as quartz, feldspars, chlorite, and metal-oxides identified by SEM/EDX exhibited corresponding Raman signals. However, some particles assigned to mica, illite, and smectite had Raman spectra that differed somewhat from those of corresponding standard minerals. Moreover, some particles identified as mixed-layer clays that could not be identified by SEM/EDX exhibited RMS spectra aligned with standard mica, illite, smectite, amphibole, pyroxene, and zeolite. This discrepancy between SEM/EDX- and RMS-based mineral assignments was likely due to the substitution and/or depletion of chemical elements because of chemical weathering^26^, which also made it difficult to identify these minerals using SEM/EDX data. In addition, all particles identified as kaolinite and pyrophyllite produced F/D–G signals, nonetheless, these particles were identified unambiguously by SEM/EDX. Consequently, mineral particles assigned to mica, illite, smectite, and mixed-layer clays were classified as a composite group of ‘other (alumino)silicates’ in this study. Thus, we examined mineralogical compositions consisting of quartz, feldspars, kaolinite, pyrophyllite, chlorite, and metal-oxides, as determined by SEM/EDX, and focused on mineralogical features to constrain changes in the major dust source regions across the NEEM LGM record.

### Mineral compositions

The most abundant mineral component found in all samples was quartz, which accounted for 21.5 ± 5.1% (min–max: 13.9–32.6%) (Table 1). Feldspars were the second most abundant mineral with an abundance of 14.0 ± 3.1% (9.7–18.1%) followed by kaolinite and chlorite at 3.5 ± 1.9% (1.5–7.3%) and 3.6 ± 1.6% (1.5–6.2%), respectively. Samples contained negligible levels of pyrophyllite, and (Fe,Ti)-rich oxides accounted for only 0% to 3.6% (mean ± SD: 1.4 ± 1.3%). On the other hand, the mean abundance of other (alumino)silicates was relatively high at 56.0 ± 7.3% (43.3–68.0%).

The mineral compositions of our samples were similar to those reported by Maggi^13^ for the Greenland Ice Core Project (GRIP) ice core LGM dust, who determined mean ± SD values of 26.2 ± 7.1% for quartz, 16.8 ± 2.4% for feldspars, 0.8 ± 1.5% for kaolinite, 2.3 ± 2.2% for chlorite, and 1.7 ± 2.3% for metal-oxides, and to those reported by Stoll et al.^27^ for two EastGRIP (EGRIP) ice core samples, corresponding to GS-2.1a and GS-2.2, respectively, who determined compositions of 21.5 ± 4.7% for quartz, 15.4 ± 1.9% for feldspars, and 4.9 ± 0.1% for metal-oxides in insoluble dust particles. Note that Maggi^13^ reported the mineral compositions determined in nine GRIP LGM ice samples, consisting of three samples for GS-2.1a, two for GS-2.1b, one for GS-2.1c, and three for GS-3, which is identical to the time periods investigated in this study. Notably, the mineral compositions of GRIP LGM samples, corresponding to GS-3, reported by Svensson et al.^14^, differed considerably from those of our NEEM GS-3 samples. Note that we converted ice core chronologies relevant to previous studies to the new GICC05 timescale following Rasmussen et al.^19^ and Seierstad et al.^28^. Svensson et al.^14^ showed ~ 1.5, 7.6, and 4.6 times higher and 2.8 times lower average contents of quartz (31.3 ± 3.4%), kaolinite (13.1 ± 1.3%), chlorite (16.5 ± 1.3%) and feldspars (5.0 ± 0.8%), respectively, compared to those (quartz: 20.1 ± 5.6%, feldspars: 14.0 ± 4.2%, kaolinite: 1.7 ± 0.3%, and chlorite: 3.6 ± 1.1%) reported for NEEM GS-3 samples. This may have been largely due to differences in the analytical methods (single particle SEM/EDX versus semiquantitative bulk X-ray diffraction (XRD)) and mineral identification methods used (determination from EDX elemental compositions versus XRD semiquantitative determination)^14^. Therefore, caution should be taken when comparing our data directly with the results of earlier studies.

### Provenance-related changes in mineralogical characteristics

**Figure 1:**
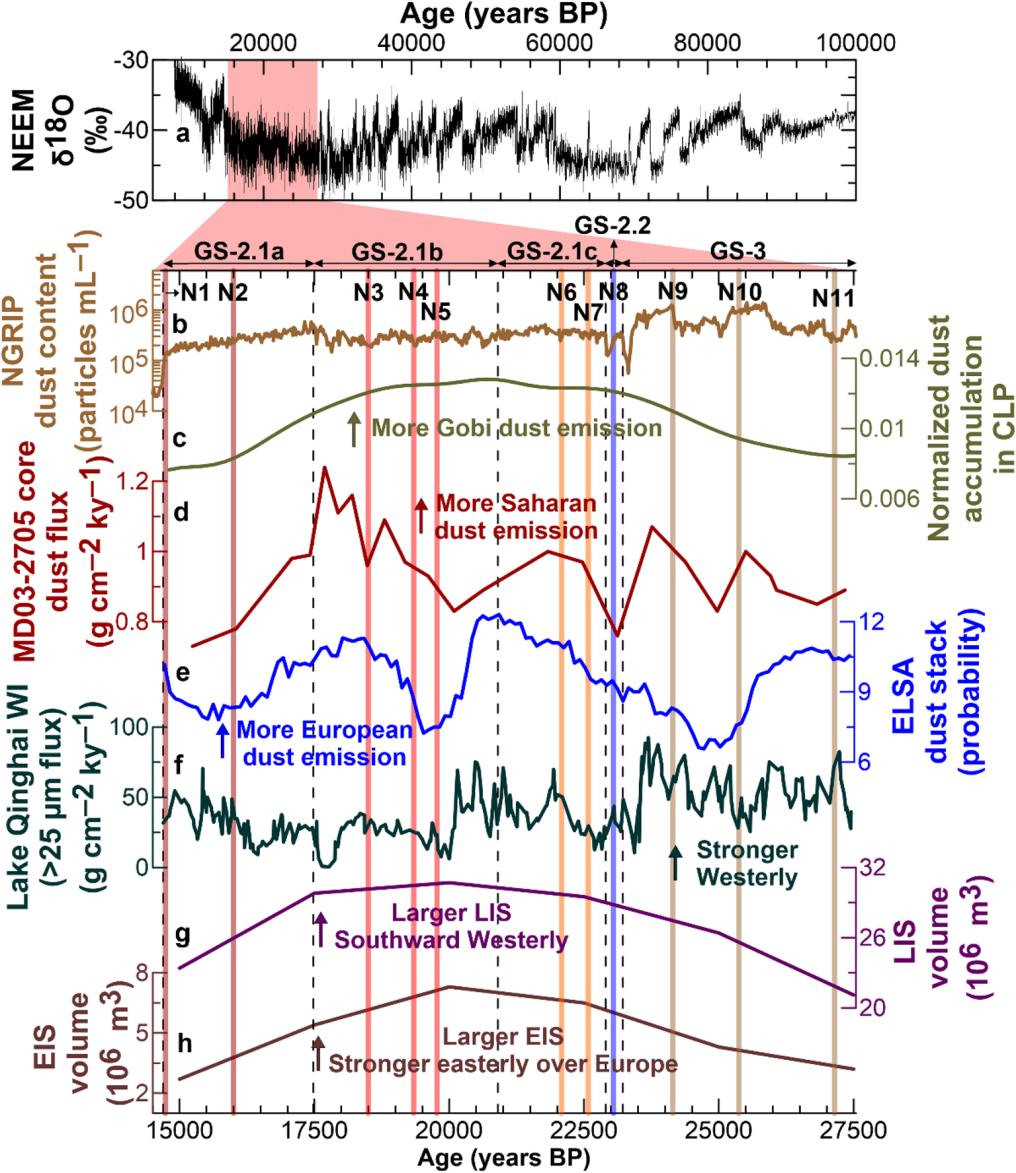
Profiles of different types of climate proxy records. (**a**) NEEM δ^18^O (Greenland temperature proxy) isotopic profile over the past 100 ky^82^ and (**b**) the NGRIP ice core dust concentration record^8^. Geenland climatic events of Greenland Stadials (GS)^19,28^ are shown at the top of the panel (**b**) and by the vertical dashed lines (see text and Table 1). Also shown are sample numbers (N1‒N11) in panel (**b**). (**c**) Dust accumulation rate record in the Chinese Loess Plateau (CLP), based on a probability density function built from 159 loess optically stimulated luminescence ages of loess in the CLP^47^. (**d**) ^230^Th-normalized Saharan dust flux record from sediment core MD03-2705 off West Africa (18° 05′N, 21° 09′W)^49^. (**e**) The Eifel Laminated Sediment Archive (ELSA) dust stack representing past dust accumulation in the Eifel region, Germany^50^. (**f**) Westerlies climate index (WI, flux of > 25 μm fraction) from Lake Qinghai (36.32°–37.15°N, 99.36°–100.47°E), located on the northeastern Tibetan Plateau^54^. (**g**,**h**) Reconstructed ice volumes of the Laurentide Ice Sheet (LIS) and Eurasian Ice Sheet (EIS), respectively^58^.

The most pronounced sample-to-sample variations in mineral contents were observed for quartz and feldspars (Table 1). The combined proportion of quartz and feldspars was the highest (49.7%) in sample N3, corresponding to GS-2.1b, and the lowest (23.7%) in sample N9, corresponding to GS-3, during which dust flux reached its maximum^8^ (Fig. 1b). Temporal variations in silicate mineral compositions were also observed in the GS sequence of GRIP LGM ice samples^13^; lower contents were observed during GS-3 and higher contents during GS-2.1a to GS-2.1b. Although it is difficult to identify the causes of changes in mineral compositions during different LGM climate periods, marked stratigraphic variations in the mineral compositions of Greenland ice core samples may indicate changes in the relative contributions of major dust source regions to Greenland dust input^10,25^. However, the size-dependent effect on mineral abundances in eolian dust must be considered to explain temporal variations in mineral compositions. For example, the abundances of quartz and feldspars in dust tend to increase with particle size^29,30^. Our results also demonstrate that the contents of quartz and feldspars significantly increased with average particle size (Pearson’s correlation coefficients 0.79 and 0.77, respectively, *p* < 0.01) (Supplementary Fig. S2). This indicates that the abundances of quartz and feldspars in samples were primarily controlled by the grain size distribution, as was previously reported for Greenland ice core dust^14,25^.

In order to further examine potential provenance-related changes in mineralogical signatures, we combined SEM/EDX with RMS results for two samples, N3 and N9 (corresponding to GS-2.1b and GS-3, respectively), which exhibited a marked difference in silicate mineral abundance (see above). As shown in Supplementary Fig. S1, mineral compositions were identified in 67.1% and 45.6% of N3 and N9 particles, respectively, and Raman F/D–G signals were detected in 26.2% and 48.9% of other (alumino)silicate particles, respectively. In addition, some particles (4.8% for N3 and 5.2% for N9) produced Raman peaks that did not match those of known minerals. F/D–G signals originate from organic carbon in soils, such as humic or humic-like substances, bound to mineral surfaces or intercalated between silicate layers^31,32^. Carbon contents determined by SEM/EDX were almost three times lower for N3 than N9 (Table 1), which supported the effect of carbon content on Raman F/D–G signals. Previous studies have reported that carbon contents in Asian deserts and European loess deposits were about 5 to 20 times greater than that in the Saharan dust during the LGM^33^. Thus, observed differences between the Raman F/D–G spectra and carbon contents of samples N3 and N9 suggest millennial-scale changes in dominant dust source, with a significantly greater contribution of Saharan dust during GS-2.1b and East Asian deserts and/or European loess deposits during GS-3. This result is supported by the higher mean carbon content of 11.3 ± 4.4% (range: 6.2–14.1%) for GS-3 samples (N9–N11) than for GS-2.1 samples (N1–N7) (mean: 7.1 ± 3.6%, range: 3.2–11.9%) (Table 1).

Subsequently, we compared kaolinite and chlorite abundance ratios (K/C ratios), which provide a valuable indicator of the source of glacial dust in Greenland ice cores^12–14^. This discriminative approach is based on the typically low K/C ratios (< ~1) of Asian deserts and European loess deposits and higher ratios (> ~1) for the Sahara^34–36^. The K/C ratios of our samples showed higher K/C values of 1.4 ± 0.7 (range: 0.8–2.8) during GS–2.1 (N1–N7) and lower values of 0.5 ± 0.1 (range: 0.3–0.6) during GS-3 (N9–N11) (Table 1). Low K/C ratios during GS-3 are consistent with those (mean ± SD: 0.6 ± 0.2; min–max: 0.3–0.8) observed in the GS-3 samples of GRIP and GISP2 (Greenland Ice Sheet Project 2) ice cores^12,14^. In contrast, Maggi^13^ found low K/C values during the GS-2.1 and GS-3 periods in GRIP ice samples. This discrepancy may be due to the different SEM/EDX-based mineral assignment criteria used, which is a modified method originally designed for transmission electron microscopy^37^, and/or larger uncertainties in determining K/C ratios caused by the relatively small numbers of particles sampled (~70 particles/sample) to identify minerals^13^. Overall, the K/C trend observed for our samples may reflect a significant contribution by the Sahara during GS-2.1 and of East Asian deserts and/or the European loess during GS-3, which is in line with the implications of significant differences between the two periods in terms of Raman F/D–G spectra and associated carbon contents. Interestingly, the K/C value during GS-2.2 had an intermediate value (0.9), suggesting mixed contributions from multiple sources.

### Chemical index of alteration (CIA) values

Chemical index of alteration (CIA) values have been employed to identify the PSAs of dust in Antarctic ice cores under different climatic regimes^38,39^. However, to the best of our knowledge, this approach has not been applied to tracing the sources of dust in Greenland ice cores. CIA values have been reported to be higher for dust from the Sahara desert (75.5 ± 7.4) than for dust from East Asian sources (Gobi Desert: 63.8 ± 3.6; Taklimakan Desert: 52.4 ± 1.5; Chinese Loess Plateau: 57.5 ± 3.8; Northern China: 53.3 ± 2.4; and Ordos Plateau: 45.8 ± 6.6) and European loess (Carpathian Basin: 64.0 ± 1.6) (Supplementary Table S1 and Fig. S3), suggesting an intensive degree of chemical weathering in the Sahara^40,41^. Thus, by using differences between the CIAs of geographical regions, we inferred the major provenances of LGM dust in Greenland ice using the CIA values of our samples.

**Figure 2:**
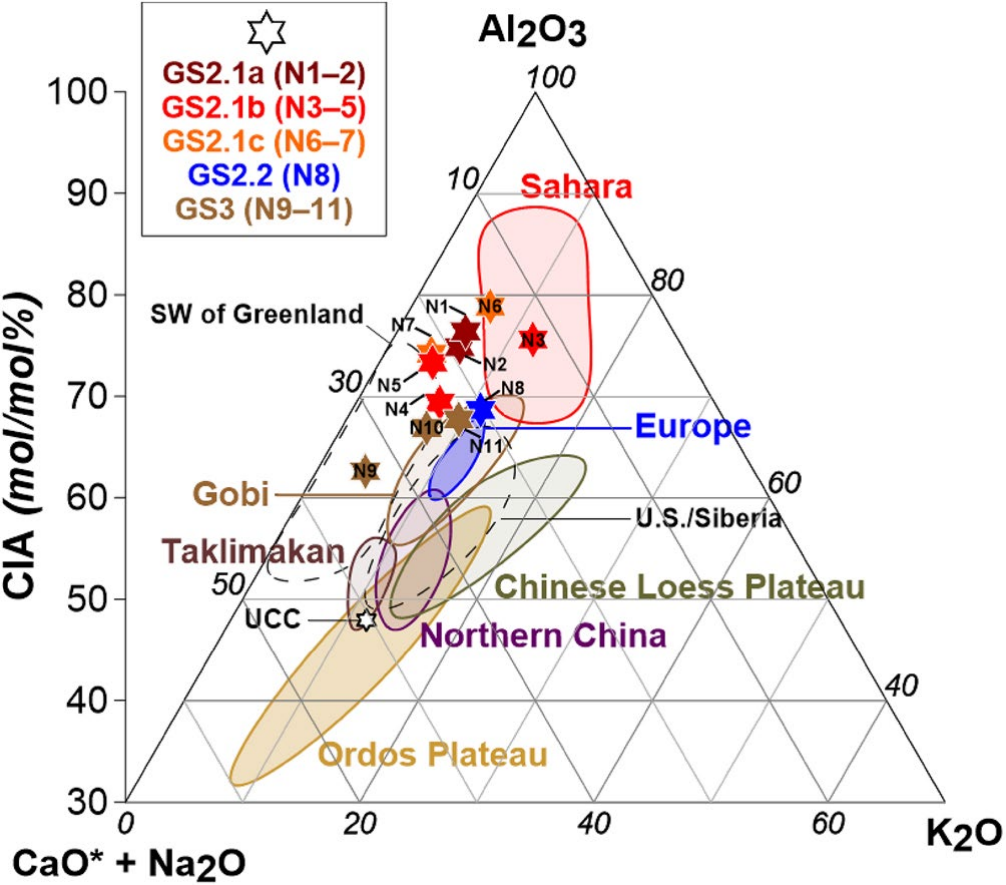
A-CN-K ternary diagram in molecular proportions with CIA values of PSAs from published literature (see Supplementary Table S1): the Sahara desert (red field); European loess deposits (blue field); the Gobi Desert (brown field); the Taklimakan Desert (dark brown field); Chinese Loess Plateau (olive field); northern China (purple field); Ordos Plateau (gold field); North American and Siberian loess deposits and southern West Greenland (dashed fields); and upper continental crust (UCC, white diamond) (see Supplementary Fig. S3). CIA values of NEEM ice core samples (Table 1) are shown in different colored stars.

CIA values of NEEM ice core samples ranged from 62.6 to 78.9 during GS-2 to GS-3 (Table 1 and Fig. 2). CIA values are significantly higher than average (47.9) for the upper continental crust^42^, indicating that particles have experienced intermediate chemical weathering. Higher CIA values (mean ± SD: 74.7 ± 2.9; min–max: 69.4–78.9) were observed for GS-2.1 samples (N1–N7), and these values were close to those of the Sahara, likely reflecting a potential Saharan contribution to Greenland dust during GS-2.1. Meanwhile, GS-3 samples (N9–N11) had a lower mean value of 65.8 ± 2.8 (min–max: 62.6–67.8) (Table 1), which was similar to those of the Gobi Desert and European loess. This suggests that Gobi, European loess, or a mixture of the two might have contributed to Greenland dust during GS-3. These results support previous findings that glacial Greenland dust during this climatic period might have originated from East Asian deserts^12,14,16,43^ and/or European loess^17,18^. Interestingly, the GS-2.2 sample N8 had an intermediate value (68.7), as did its K/C value (Table 1), probably because of dust contributions from multiple sources. To summarize, temporal variations in CIA values may reflect a steady increase in Saharan dust inputs to Greenland probably after GS-3 and peaking during GS-2.1. This feature is consistent with estimates of Greenland glacial dust provenance based on Pb and Sr isotopic signatures, which indicated that the Sahara was an important source, particularly during GS-2.1b^16^.

In Fig. 2, CIA values were plotted in the ternary diagram of Al_2_O_3_–(CaO* + Na_2_O)–K_2_O) (A–CN–K) to evaluate the trends in degrees of silicate weathering of source areas^44^. Some samples (i.e., N1, N2, N4, N5, N7, and N9) show more depleted [CaO* + Na_2_O] values than PSAs, possibly due to insufficient published data for PSAs and/or changes in the chemical compositions of dust during long-range atmospheric transport, which can cause excessive removal of calcium and sodium relative to potassium^45,46^. However, the residence times of dust in the atmosphere are much shorter than the timescales of mineral weathering in source areas, which likely limits the effect of atmospheric transport on CIA values. Thus, we believe that the variability in dust CIA values shown by samples is probably related to changes in the primary dust sources deposited in Greenland from GS-2 to GS-3.

### Possible mechanisms of observed changes in major dust sources

The dominance of source-specific dust in Greenland glacial ice might have been driven by variations of dust emissions at PSAs and/or changes in the atmospheric transport processes associated with large-scale atmospheric circulation variability during the different glacial stages. In Fig. 1, we compared NEEM δ^18^O and NGRIP dust profiles with different paleo-proxy records to infer mechanisms that contributed to the observed shift in dominant dust provenance between GS-2.1 and GS-3.

Initially, we investigated high-resolution dust flux records related to changes in the relative intensities of dust emissions over the Gobi, Sahara, and European loess over the study period. Relative variations in the mass accumulation rates of eolian deposits on the Chinese Loess Plateau (CLP)^47^, reflecting fluctuations in dust emissions from the Gobi Desert^48^, show enhanced dust emissions in the Gobi Desert over the GS-2.1 period (Fig. 1c). Interestingly, dust flux record of marine sediments off West Africa^49^, referred to as an imprint of Saharan dust emission variability (Fig. 1d), also revealed a noticeable rise in dust emissions over the Sahara during GS-2.1, particularly during GS-2.1b, albeit with some fluctuations. Subsequently, dust emission rates from European loess deposits, obtained from the ELSA (Eifel Laminated Sediment Archive) records in central Europe (Eifel, Western Germany)^50,51^ (Fig. 1e), were characterized by significant variations and greater dust emission during GS-2.1, particularly from GS-2.1b to GS-2.1c. In summary, these patterns suggest that dust transport efficiency may have played an important role in the dominance of dust supply from the Gobi, Sahara, and European loess to Greenland in the LGM rather than changes in dust emissions from these source regions.

Because East Asian dust that reach the Arctic is primarily delivered by the prevailing Westerlies in the mid-latitudes of the Northern Hemisphere^52,53^, we then examined the relative intensities of Westerlies during GS-2.1 and GS-3. For this, we used a Westerlies climate index (WI) as indicated by variations in the content of the > 25 μm fraction of sediments in the Westerlies-influenced Lake Qinghai on the northeastern Tibetan Plateau^54^ (Fig. 1f). Large WI values indicate the strengthened influence of Westerlies and intensified aridification over inland East Asia. WI values were lower during GS-2.1 than during GS-3, although they fluctuated over the entire time series (Fig. 1f). Hence, we infer that weakening of Westerlies during GS-2.1 may have been a cause of reductions in Gobi contributions to Greenland dust deposition during this period. Compared with the transport pathway of East Asian dust, Saharan dust plumes can directly reach central Greenland by accompanying a westward movement to the Atlantic Ocean by trade winds and a subsequent northward movement by Westerly winds passing through the subpolar North Atlantic driven by low-pressure systems^55,56^. Previous studies have suggested a more southward shift and greater intensification of North Atlantic Westerlies during GS-2.1 than GS-3, potentially as a result of the southward migration of the deeper Icelandic Low in association with a larger volume of the Laurentide Ice Sheet (LIS) during this period^57–59^ (Fig. 1g). Furthermore, such atmospheric circulation changes may have caused an increase in dust transport from the Sahara to Greenland during GS-2.1.

Recently, Li et al.^43^ proposed that LIS growth may have led to the division of Westerly winds into northern and southern branches over the LIS. The northern branch would have provided a faster transport pathway from East Asian deserts to Greenland and hence reduced en route dust losses, which, in turn, may have increased the transport of Asian dust to Greenland and likely increased Greenland dust concentrations^9,43^. However, the northern branch was comparatively weaker than the southern branch^60,61^, and the NGRIP dust record shows higher (lower) dust content levels during the GS-3 (GS-2.1) period^8^ (Fig. 1b), characterized by smaller (larger) LIS volume (Fig. 1g) and stronger (weaker) Westerlies (Fig. 1f). Given these findings, it is more likely that the larger LIS during GS-2.1 may have led to a stronger impact on the North Atlantic atmospheric circulation, which eventually facilitated more frequent Saharan dust transport to Greenland during this period and a substantial reduction in the East Asian contribution.

Finally, we examined the potential contributions of dust from European loess deposits associated with variations in atmospheric circulation patterns during GS-2.1 and GS-3. During the LGM, dust from European loess deposits might have been transported to the North Atlantic by easterly winds induced by anticyclonic systems over the Eurasian Ice Sheet (EIS)^62,63^ and then to Greenland by low-pressure systems over the North Atlantic^17^. As shown in Fig. 1h, the EIS was at its maximum extent and volume during GS-2.1b^58^ and likely caused stronger anticyclonic flow and associated easterlies due to a maximum katabatic wind speed over the EIS^64^, which may have facilitated the supply of dust from European sources toward Greenland during the GS-2.1 period. However, our mineralogical and geochemical data records demonstrate a dominant contribution of Saharan dust during GS-2.1, probably because of the occurrence of more complex anticyclonic near-surface wind patterns over Europe when the topographic height and extent of the EIS increased significantly^64^, ultimately resulting in less favorable conditions for transporting European dust to the North Atlantic. Meanwhile, the mineralogical and geochemical signatures of our samples suggest a potential contribution of dust from European loess to Greenland dust deposition during GS-3, which is consistent with the results of Újvári et al.^17^. As such, it is plausible that the more effective atmospheric conditions for European dust transport toward Greenland occurred during GS-3 when EIS volume was smaller (Fig. 1h). This situation suggests the presence of internal atmospheric circulation variability over Europe during GS-2.1 and GS-3, which may have resulted in increased cyclone frequency in winter and more intense southerly flow pattern in central Europe under colder climatic conditions during GS-3^65^. Additional high-resolution proxy data and climate model studies are required to establish a more detailed understanding of the main drivers of millennial-scale variability in the relative contributions of individual sources to dust deposited in Greenland during the LGM.

## Conclusion

This study provides data on the morphological, mineralogical, and geochemical characteristics of insoluble particles in Greenland NEEM ice core samples from GS-2.1a to GS-3 (~ 14.7 to 27.1 kyr ago) during the LGM as obtained using SEM/EDX and Raman spectroscopy techniques on a single particle basis to gain insights into millennial-scale variability in Greenland glacial dust provenance. The results show that physical (size and morphology) and mineralogical properties do not provide important constraints on the primary source of Greenland dust. In contrast, we observed considerable differences in the geochemical characteristics of particles at different times during the LGM. GS-2.1a to GS-2.1c samples had higher K/C ratios (1.4 ± 0.7), lower carbon contents (7.1 ± 3.6%), and higher CIA values (74.7 ± 2.9) than GS-3 samples (K/C: 0.5 ± 0.1, carbon contents: 11.3 ± 4.4%, CIA: 65.8 ± 2.8). These geochemical signatures were probably associated with shifts in dominant dust sources and larger contributions of Saharan dust during GS-2.1 and Gobi and/or European loess dust during GS-3, which is consistent with previous estimates of proportional dust source contributions obtained using a mixing model based on Pb and Sr isotopic compositions in NEEM LGM ice. A comparison of our results with different paleo-proxy records suggests that the appearance of millennial-scale dust source changes in Greenland LGM glacial ice may have been linked to large-scale atmospheric circulation variability associated with the evolution of continental-scale ice sheets (the Laurentide and Eurasian Ice Sheets) throughout the LGM. It would be interesting to investigate millennial-scale variability of Greenland dust provenance for the last glacial period characterized by abrupt climate changes.

## Materials and methods

### Ice core samples and the decontamination procedure

We selected 11 core sections from the 2540-m-long NEEM deep ice core drilled in Northwest Greenland (77.45° N; 51.06° W; 2450 m above sea level)^66^ (Supplementary Fig. S4). Sample depths ranged between 1490.15 and 1633.15 m, which corresponded to the ages of ~ 14,737 to 27,144 years before 2000 CE (b2k), respectively^67^ (Table 1). Seven samples were selected for GS-2.1a to GS-2.1c, one for GS-2.2, and three for GS-3 in the LGM (Table 1). Note that the sequence of GS is defined as the cold periods based on the robust correlation between different proxy climate records from three synchronized Greenland ice cores^19^.

Each ice section (20 cm in length) had a cross-section of 4 × 4 cm^2^ and was mechanically decontaminated to remove the outside of the core using ultraclean procedures, as described by Han et al.^68^. Briefly, each ice section was secured in an acid-cleaned cylindrical Teflon tumbler holder by Teflon screws and decontaminated by chiseling three successive veneer layers of ice in progression from the outside to the innermost part to obtain an uncontaminated inner part of the section. Chiseling was performed inside a class 10 vertical laminar flow clean bench at − 15 °C using acid-cleaned stainless-steel chisels (custom-made with a single plate of 2 mm thick stainless-steel type 316 L) or ceramic knives (Kyocera Advanced Ceramics, Models: FK075WH) at the Korea Polar Research Institute (KOPRI), Korea. Inner core samples were divided into two pieces (each 10 cm long), which were recovered directly into ultraclean 1 L wide mouth low-density polyethylene (LDPE) bottles (Nalgene, Thermo Fisher Scientific, Germany) and stored frozen until analysis. Upper pieces, spanning approximately 2 years of snow accumulation (Table 1), were used for this study.

### Sample preparation for single particle analysis

**Figure 3:**
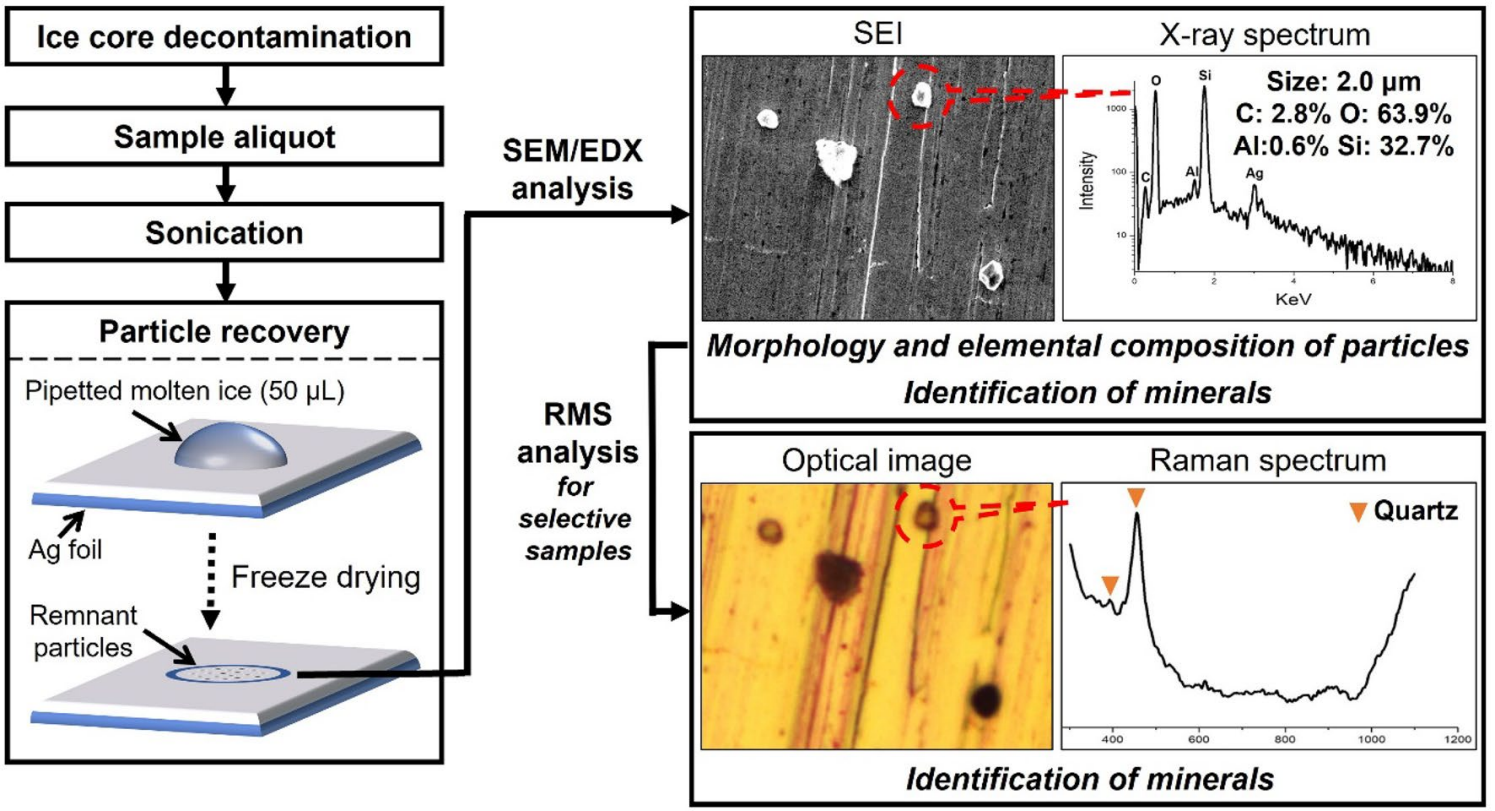
A general scheme for retrieving insoluble particles in Greenland NEEM ice core samples and the combination of SEM/EDX and RMS for a detailed physiochemical characterization of individual particles on a single particle basis.

Ice core samples were first melted at room temperature in a class 10 clean bench inside a non-laminar flow class 1000 clean laboratory at KOPRI. The samples were then shaken to distribute particles homogeneously, aliquoted into precleaned 15 mL polypropylene conical centrifuge tubes (Corning, USA), and sonicated in an ultrasonic bath (Powersonic 620, Hwashin Technology, Republic of Korea) for 1 h to break particle aggregates (Fig. 3), possibly formed by post-depositional processes in the ice matrix^69^.

A microdroplet (50 μL) of each sample was loaded on silver (Ag) foil of ~ 1 × 1 cm^2^ (99.95% purity, 0.025 mm thickness; Goodfellow Inc., UK) attached to an aluminum stub using a micropipette (Fig. 3). Sample sizes were designed to load sufficient amounts of particles on the Ag foil based on particle concentrations during the LGM (~ 2–3 × 10^5^ particles mL^−1^) as measured in the NGRIP ice core^8^. Before using Ag foils, surface cleanliness was checked using a SEM (Supplementary Fig. S5a) and foils were immersed in liquid nitrogen for a minute to prevent potential reactions between them and chloride species in ice samples. Note that although non-reactive aluminum (Al) foil can be used as a potential substrate, we preferred to use Ag foil to avoid Al-induced potential interference from Al substrate when calculating the CIA values (see below).

Using a Lyovapor L-200 freeze dryer (Buchi Labortechnik AG, Switzerland), samples were finally freeze-dried within an hour at a condenser temperature of − 56 °C and chamber pressure of 0.5 Mbar. These conditions allowed the deposition of individual particles on Ag foils due to the sublimation of water (Fig. 3). The freeze dryer was equipped with a needle valve to regulate airflow, which prevent particles from scattering on foils after sublimation. Note that water-soluble compounds were almost nonexistent on Ag foils after freeze-drying and that those remaining had smaller particle sizes (submicron to nano-sized) than the larger insoluble particles.

Procedural blanks were determined by loading ultrapure water droplets from a Milli-Q Integral water purification system (Merck Millipore, USA) onto Ag foils in an identical manner. The maximum number of particles on a single foil substrate was restricted to one (Supplementary Fig. S5a–c), which guaranteed effective control of particulate contamination.

### SEM/EDX and RMS measurements

The morphology and elemental compositions of individual particles in the 11 NEEM samples were determined by SEM/EDX, which was conducted using a JEOL JSM-6390 SEM instrument equipped with an Oxford Link SATW ultrathin window EDX detector with a 133 eV spectral resolution for Mn Kα X-rays. The operating conditions used to obtain secondary electron images (SEIs) and X-ray spectra of individual particles were accelerating voltage 10 kV, beam current 1.0 nA, and measurement time 20 s. X-ray spectra were processed using Oxford INCA Energy software, and EDX data were obtained using a microanalytical unit that provided the chemical composition of particles. Net X-ray intensities of elements were obtained using a non-linear least-squares method in the AXIL program^70^, and elemental concentrations were determined from X-ray intensities by Monte Carlo calculation combined with reverse successive approximations^71,72^. Determined elements involved low-Z (C and O) and major (Na, Mg, Al, Si, K, Ca, Ti, and Fe) elements. The EDX software normalizes analytical totals of their atomic fractions to 100%. As such, the analytical totals below 97% were rejected to minimize the analytical errors. This quantification procedure yielded accurate results with a relative deviation of ≤ 12% when applied to various types of standard particles^72^. SEM/EDX analysis was performed on 187–200 particles per sample (Table 1), which was sufficient to characterize discrete distributions in a particle population^73^.

RMS analysis was also conducted to identify particle mineral phases in two selected ice samples (N3 and N9 in Table 1) after relocating the particles using the RMS optical images (Fig. 3). This analysis was performed using a confocal micro-RMS system (XploRA, Horiba-Scientific, France) equipped with a microscope (BX41, Olympus, Tokyo, Japan) and a liquid-nitrogen-cooled charge-coupled detector. Using a 100 × objective with a 0.9 numerical aperture at 638 nm laser excitation wavelength, a laser beam of ~ 11 mW was focused to a spot area of ~ 1 µm^2^. Raman spectra were collected per sample from 100 to 3000 cm^−1^ with an acquisition time of 10 s and five-time accumulation per analysis. Spectral analysis was conducted using Labspec 6.0 software. As shown in Fig. 3, this combined X-ray/Raman spectroscopic approach was used to confirm the mineralogy of particles^31^.

In Supplementary Section S2, we present the SEM/EDX and Raman spectroscopy results of single particles subjected to different sample preparation procedures. These procedures failed to produce successful results for the mineralogical and chemical characteristics of particles, which showed that initial testing is required to confirm that the intended procedure enables the accurate characterization of individual insoluble particles using spectroscopic techniques when available sample volumes are limited to micrometer-sized droplets.

### Mineral identification

Initially, we compared the major elemental compositions of individual particles determined by SEM/EDX with the calculated chemical formulars of standard minerals^13,24,25,37^, allowing the discrimination and identification of minerals for each particle in the samples. According to the compositions and distributions of elements obtained by the results of X-ray spectral analysis, quartz (SiO_2_) was characterized by high Si and O contents with no discernible impurities, while feldspars (KAlSi_3_O_8_, NaAlSi_3_O_8_, CaAl_2_Si_2_O_8_, or feldspar mixtures) had Si values up to ~ 3 times higher or similar to those of Al, K, Na, and/or Ca. Kaolinite (Al_2_Si_2_O_5_(OH)_4_) and pyrophyllite (Al_2_Si_4_O_10_(OH)_2_) were distinguished by Si/Al ratios of ~ 1 and 2, respectively, and no discernible impurities. The metal-oxides minerals (e.g., (Fe, Ti)–oxides) contained high amounts of Fe or Ti with trace levels of other elements.

Although the abovementioned minerals could be characterized and identified by their elemental compositions, some aluminosilicates such as chlorite, mica (e.g., muscovite), illite, smectite (e.g., montmorillonite), and mixed-layer clays were difficult to differentiate based on element distributions, because they contain a variety of substitutable elements. Therefore, for these aluminosilicates, a peak ratio sorting scheme based on elemental ratios was used for mineral identification (see Fig. 2 in Donarummo et al.^24^). This approach was shown to be useful for identifying the mineralogy of dust in Greenland ice cores^13,24,25^.

To aid SEM/EDX-based mineral identification, SEM/EDX and RMS methods were combined to identify specific mineral species in particles in samples nos. N3 and N9. The online RRUFF database (https://rruff.info) and all published Raman spectral data^74–77^ were used for mineral identification based on Raman analysis. Typical SEM/EDX and Raman spectra of the different minerals found in the two core samples are shown in Supplementary Figs. S6 and S7, respectively.

### CIA index

CIA was first proposed by Nesbitt and Young^44^ as a proxy for the chemical weathering of soils and sediments in source areas and has been widely used to provide information on source characteristics and the provenance of terrestrial materials utilized for paleoclimate reconstruction^44,78,79^. High CIA values reflect the preferential removal of mobile cations (e.g., Ca, Na, and K) relative to refractory elements (e.g., Al and Ti) during chemical weathering. In contrast, low CIA values represent the almost complete absence of chemical alteration^44^.

CIA values were defined as the molar ratio of immobile metal oxides to mobile metal oxides using the following equation:

$$ {\text{CIA}} = \left[ {{\text{Al}}_{{2}} {\text{O}}_{{3}} /\left( {{\text{Al}}_{{2}} {\text{O}}_{{3}} + {\text{CaO}}* + {\text{Na}}_{{2}} {\text{O}} + {\text{K}}_{{2}} {\text{O}}} \right) \times {1}00} \right] $$

where CaO* represents the amount of CaO in the silicate fraction only. Major elemental compositions determined by SEM/EDX were used to calculate CIAs by converting the concentrations of chemical species in individual particles to total molar fractions of major elements in samples (for more details, refer to Supplementary Section S3). Note that the chemical compositions of individual particles in our samples were measured only for silicate materials, and thus, it was not necessary to correct measured CaO concentrations for CaO*. For comparison, we calculated CaO* for the bulk chemical compositions of PSAs by quantifying CaO contents in silicate fractions and assuming a reasonable Ca/Na ratio in silicate materials^78^.

As the chemical compositions of dust particles with different particle sizes can vary greatly, that is, as particle size decreases, Al_2_O_3_, Fe_2_O_3_, and K_2_O contents increase, and SiO_2_ and Na_2_O contents decrease^29,80^, the PSA dataset used in this study was derived from size-segregated elemental composition data extracted from available literature to reduce the ‘grain size effect’ on CIA values (see Supplementary Table S1 and Fig. S3). In addition, we used the oxide concentration dataset from soil and airborne dust aerosol measurements in or from western North Africa (e.g., Algeria, Mali, Mauritania, Morocco, and Senegal) to represent Saharan dust (Supplementary Table S1) because Saharan dust that travels to Greenland across the Atlantic Ocean originates primarily from this region^81^.

### Supplementary Information

Supplementary Information.

## Data Availability

All the data used in this study can be obtained from the tables presented in the main text and the Supplementary Information.
