# Supplementary material for: Millennial-scale variability of Greenland dust provenance during the last glacial maximum as determined by single particle analysis
Source: Sci Rep. 2024 Jan 23;14:2040. doi: 10.1038/s41598-024-52546-x (PMC10805741; doi:10.1038/s41598-024-52546-x)
Supplement: Supplementary file 1 — Supplementary Information. [file 41598_2024_52546_MOESM1_ESM.docx]

**Supplementary Information**

**Millennial-scale variability of Greenland dust provenance during the Last Glacial Maximum as determined by single particle analysis**

Seokhyun Ro^a,b,†^, Jonghyeon Park^c,d,†^, Hanjin Yoo^c,e^, Changhee Han^f^, Ahhyung Lee^a,g^, Yoojin Lee^c^, Minjeong Kim^b,c^, Yeongcheol Han^b^, Anders Svensson^h^, Jinhwa Shin^b^, Chul-Un Ro^c,e,*^, and Sungmin Hong^a,**^

^a^Department of Ocean Sciences, Inha University, 100 Inha-ro, Michuhol-gu, Incheon 22212, Republic of Korea

^b^Division of Glacial Environment Research, Korea Polar Research Institute, 26 Songdomirae-ro, Yeonsu-gu, Incheon 21990, Republic of Korea

^c^Department of Chemistry, Inha University, 100 Inha-ro, Michuhol-gu, Incheon 22212, Republic of Korea

^d^Marine Environment Research Department, Ara Consulting and Technology, 30 Songdomirae-ro, Yeonsu-gu, Incheon 21990, Republic of Korea

^e^Particle Pollution Research and Management Center, Inha University, 36 Gaetbeol-ro, Yeonsu-gu, Incheon 21999, Republic of Korea

^f^Department of Water Environmental Safety Management, Korea Water Resources Corporation, 200 Sintanjin-ro, Daedeok-gu, Daejeon 34350, Republic of Korea

^g^Unit of Frontier Exploration, Korea Polar Research Institute, 26 Songdomirae-ro, Yeonsu-gu, Incheon 21990, Republic of Korea

^h^Centre for Ice and Climate, Niels Bohr Institute, University of Copenhagen, Julian 10 Maries Vej 30, 2100 Copenhagen, Denmark

^†^These authors contributed equally to this work and share first authorship

^*^Corresponding author: *Email address:* curo@inha.ac.kr (Chul-Un Ro).

^**^Corresponding author. *Email address:* smhong@inha.ac.kr (Sungmin Hong).

**Section S1. Statistical relationships between particle diameter and aspect ratio**

We performed statistical analyses to examine the relationship between mean particle size and mean aspect ratio of a given sample (see Table 1) using the Statistical Package for Social Science (SPSS, version 12.0K, IBM, USA). First, a boxplot analysis was conducted to detect the presence of outliers in the data, because outliers can distort statistical analyses and skew results. As shown in **Supplementary Fig. S8**, no outliers were detected. Then, we examined the statistical significance of temporal differences in the particle size and aspect ratio between GS–2.1 (N1-N7) and GS–3 (N9-N11) samples. Initially, the Kolmogorov-Smirnov normality test was used to determines whether the data follows a normal distribution (two-tailed *p*-value > 0.05 means in a normal distribution). The results showed that particle sizes are normally distributed (*p* = 0.200), while aspect ratios do not follow a normal distribution (*p* = 0.001). Therefore, a paired t-test was used to examine the difference between the means of particle diameter (statistically significant if *p* < 0.05). By comparison, the Wilcoxon signed-rank test was carried out to compare the aspect ratio data (statistically significant if *p* < 0.05). The results showed that the differences in the mean values of particle diameter and aspect ratio between samples were not statistically significant (p = 0.510 and 0.317, respectively). In consequence, statistical analyses do not allow us to gain meaningful insights from data to explain the short-term variations in mean particle size and aspect ratio across the LGM.

**Section S2. Unsuccessful methods used for applying SEM/EDX and RMS analyses to individual ice core particles loaded on a substrate**

*S1.1. Microdrop deposition technique*

The microdrop deposition technique, as outlined by Murr et al.^1^ and Paleari et al.^2^, involves loading insoluble ice core particles on substrates by pipetting molten ice sample droplets and then drying. Here, we replicated this procedure by evaporating a 30 μL droplet of ultrapure water containing a powdered kaolinite standard reference material (SRM) (CAS No. 1318–74–7, Sigma-Aldrich, USA) of < 25 μm in size, on a silver (Ag) foil (99.95% purity, 0.025 mm thickness; Goodfellow Inc., UK). This evaporation was performed at room temperature under a clean bench (class 10) and in an oven set at 100˚C. However, remaining particles smaller than 5 μm accumulated at the droplet perimeter^3^ due to the so-called “coffee–stain effect^4^” (**Supplementary** **Fig. S9**). Although some particles were found at the droplet centers (**Supplementary** **Fig. S9b**), they typically had diameters exceeding 10 μm, likely resulting from the faster settling of larger particles. Given the typical diameter of Greenland dust (~1.7–2 μm)^5,6^, use of the microdrop deposition technique for single particle analysis of such ice core particles is challenging.

*S1.2. Filtering method*

The filtering method has been traditionally used for single particle analysis of insoluble ice core particles^7–9^. This method involves filtering molten ice samples through polycarbonate filters. In this study, we replicated the method by filtering SRM kaolinite particle-containing ultrapure water through a gold (Au)-coated polycarbonate filter (13 mm diameter, 0.4 μm pore size; Sterlitech Corporation, USA). The method was confirmed to be applicable for both SEM/EDX and RMS measurements on same SRM particles. SEM/EDX operating conditions were accelerating voltage 10 kV, beam current 0.8 nA, and measuring time 20 s. Raman scattering was performed at a laser wavelength of 638 nm at a delivered power of ~3 mW. More information on these analytical setups is provided in the main text.

However, while SEM/EDX was successfully applied to measure the ‘real’ ice core particles loaded on a substrate using this filtering method, difficulties were encountered for RMS measurements as most of the analyzed particles failed to produce sufficient mineral-inherent Raman signals due to an overpowering fluorescence signal followed by the Raman D–G band (defined as the F–D/G signal, see **Supplementary** **Fig. S7**); for more detail on the occurrence of the F–D/G signal for ice core particles, see the main text), which were not observed for SRM kaolinite particles. Eliminating F–D/G signals would require more Raman laser power. However, we found that at higher power, the Au-coated polycarbonate filter was deformed (**Supplementary** **Fig. S10**). This deformation also occurred when standard polycarbonate filters were used, which rendered the filtering method unsuitable for RMS measurements on insoluble ice core particles.

**Section S3. Conversion of atomic proportions into molar proportions**

The total molar fractions were calculated by dividing total weight fractions of specific chemical elements by their corresponding molecular weights. Total weight fractions of major elements were obtained by dividing total weights of each element by the total weight of all elements in analyzed particles, which can be approximated based on the relative weights of elements in their oxide forms within each particle and the overall masses of each particle. The relative weight fractions of each major element in its oxide form were determined by multiplying its relative fraction within a particle (estimated using atomic concentrations) by the formula weight of its oxide^10^. Overall particle mass was derived by multiplying mass density by its estimated 3D-volume. A standard particle density of 2.65 g cm^‒3^, which is typical for most dust particles, was used in the calculation. Particle volumes were estimated using 2D secondary electron images (SEIs). However, approximations of particle 3D volumes have inherent limitations. In particular, particle diameters used for the 3D calculations were derived from 2D images, and particles were assumed to be spherical, although most had an irregular shape. Nevertheless, the weight fractions of major minerals in powdered potassium feldspar SRM samples determined using this single particle analytical approach agreed with certified values obtained using bulk analytical techniques within a margin of 8%^11^. Further information regarding the procedure for converting the atomic proportions into molar proportions can be found elsewhere^12^.

**Table S1.** Major elemental compositions (wt%) of the PSA from literature and calculated their CIA values (mol/mol%).

| **Reference** | **Sampling site**  **(Source area)** | **Type** | **Size** | **Fe_2_O_3_** | **MnO** | **TiO_2_** | **CaO** | **K_2_O** | **P_2_O_5_** | **SiO_2_** | **Al_2_O_3_** | **MgO** | **Na_2_O** | **CIA** |
| --- | --- | --- | --- | --- | --- | --- | --- | --- | --- | --- | --- | --- | --- | --- |
| Jiang & Wang^13^ | Taklimakan Desert,  East Asia | Sediment | < 63 µm | 4.0 | 0.1 | 0.8 | 9.6 | 1.8 | 0.2 | 61.9 | 9.9 | 2.3 | 2.1 | 52.2 |
|  |  |  |  | 4.2 | 0.1 | 0.7 | 12.2 | 1.5 | 0.2 | 57.8 | 9.2 | 2.3 | 2.0 | 53.4 |
|  |  |  |  | 4.7 | 0.1 | 1.0 | 15.2 | 1.5 | 0.3 | 51.8 | 8.8 | 3.1 | 1.8 | 53.8 |
|  |  |  |  | 3.6 | 0.1 | 0.7 | 12.8 | 1.7 | 0.2 | 56.1 | 9.1 | 2.8 | 2.0 | 52.4 |
|  |  |  |  | 4.6 | 0.1 | 1.0 | 13.9 | 1.6 | 0.3 | 52.9 | 8.9 | 3.1 | 1.9 | 53.0 |
|  |  |  |  | 4.3 | 0.1 | 0.9 | 14.0 | 1.6 | 0.3 | 53.4 | 8.9 | 3.1 | 1.9 | 52.8 |
|  |  |  |  | 3.6 | 0.1 | 0.7 | 13.4 | 1.6 | 0.2 | 55.0 | 8.8 | 2.9 | 1.9 | 52.3 |
|  |  |  |  | 3.9 | 0.1 | 0.8 | 13.3 | 1.7 | 0.2 | 54.7 | 9.0 | 2.9 | 1.9 | 52.3 |
|  |  |  |  | 4.0 | 0.1 | 0.8 | 13.6 | 1.7 | 0.2 | 54.4 | 9.0 | 3.0 | 1.9 | 52.8 |
|  |  |  |  | 3.9 | 0.1 | 0.8 | 14.0 | 1.6 | 0.2 | 54.1 | 8.9 | 3.0 | 1.9 | 52.8 |
|  |  |  |  | 4.3 | 0.1 | 0.9 | 14.4 | 1.6 | 0.3 | 52.9 | 9.0 | 3.1 | 1.9 | 53.0 |
|  |  |  |  | 4.4 | 0.1 | 0.9 | 14.4 | 1.6 | 0.3 | 52.6 | 8.9 | 3.1 | 1.8 | 53.5 |
|  |  |  |  | 4.7 | 0.1 | 1.0 | 14.4 | 1.6 | 0.3 | 52.3 | 8.8 | 3.1 | 1.9 | 52.6 |
|  |  |  |  | 3.9 | 0.1 | 0.8 | 13.1 | 1.7 | 0.2 | 55.1 | 9.1 | 2.9 | 1.9 | 52.6 |
|  |  |  |  | 4.0 | 0.1 | 0.8 | 13.9 | 1.6 | 0.2 | 53.7 | 8.7 | 3.0 | 1.9 | 52.1 |
|  |  |  |  | 3.9 | 0.1 | 0.8 | 14.1 | 1.6 | 0.2 | 53.4 | 8.9 | 3.0 | 1.9 | 52.4 |
|  |  |  |  | 4.9 | 0.1 | 1.0 | 15.5 | 1.4 | 0.3 | 49.9 | 8.5 | 3.3 | 1.7 | 53.9 |
|  |  |  |  | 4.5 | 0.1 | 1.0 | 14.3 | 1.6 | 0.3 | 52.3 | 8.8 | 3.1 | 1.8 | 53.3 |
|  |  |  |  | 4.2 | 0.1 | 0.9 | 14.7 | 1.6 | 0.3 | 52.5 | 8.9 | 3.2 | 1.9 | 53.0 |
|  |  |  |  | 4.3 | 0.1 | 0.9 | 14.3 | 1.6 | 0.2 | 52.4 | 8.8 | 3.1 | 1.9 | 52.9 |
|  |  |  |  | 5.9 | 0.1 | 1.3 | 14.9 | 1.5 | 0.3 | 49.6 | 8.7 | 3.3 | 1.8 | 54.1 |
|  |  |  |  | 4.6 | 0.1 | 1.0 | 14.3 | 1.6 | 0.3 | 52.8 | 9.0 | 3.1 | 1.8 | 53.7 |
|  |  |  |  | 4.6 | 0.1 | 1.0 | 13.9 | 1.6 | 0.3 | 53.3 | 9.0 | 3.1 | 1.9 | 52.7 |
|  |  |  |  | 6.4 | 0.2 | 1.4 | 14.8 | 1.5 | 0.3 | 49.5 | 8.8 | 3.4 | 1.7 | 54.6 |
|  |  |  |  | 4.8 | 0.1 | 1.0 | 14.4 | 1.6 | 0.3 | 51.6 | 9.0 | 3.2 | 1.9 | 53.4 |
|  |  |  |  | 6.0 | 0.1 | 1.3 | 14.6 | 1.5 | 0.3 | 49.9 | 8.8 | 3.3 | 1.8 | 54.3 |
|  |  |  |  | 4.2 | 0.1 | 0.9 | 14.3 | 1.6 | 0.3 | 52.8 | 9.0 | 3.1 | 1.8 | 53.7 |
|  |  |  |  | 4.2 | 0.1 | 0.9 | 13.1 | 1.6 | 0.2 | 54.4 | 8.9 | 2.8 | 1.9 | 52.8 |
|  |  |  |  | 3.6 | 0.1 | 0.7 | 12.4 | 1.8 | 0.2 | 56.9 | 9.3 | 2.7 | 2.0 | 52.3 |
|  |  |  |  | 4.8 | 0.1 | 1.1 | 14.4 | 1.6 | 0.3 | 52.3 | 9.0 | 3.2 | 1.9 | 53.4 |
| Yang  et al.^14^ |  |  | < 53 µm | 3.0 | 0.1 | 0.5 | 10.9 | 1.8 | 0.1 | 58.5 | 9.6 | 2.4 | 2.2 | 50.8 |
|  |  |  |  | 2.8 | 0.1 | 0.5 | 10.3 | 1.8 | 0.1 | 59.8 | 9.7 | 2.5 | 2.2 | 51.6 |
|  |  |  |  | 2.3 | 0.1 | 0.4 | 9.1 | 2.0 | 0.1 | 62.8 | 9.6 | 2.0 | 2.4 | 49.3 |
|  |  |  |  | 5.2 | 0.1 | 0.9 | 9.2 | 1.8 | 0.1 | 58.7 | 10.0 | 2.7 | 2.3 | 51.5 |
|  |  |  |  | 3.0 | 0.1 | 0.5 | 7.0 | 2.0 | 0.1 | 65.7 | 10.0 | 1.9 | 2.4 | 49.9 |
| Yang  et al.^14^ | Taklimakan Desert,  East Asia | Sediment | < 53 µm | 3.0 | 0.1 | 0.5 | 7.4 | 2.0 | 0.1 | 64.8 | 10.3 | 2.1 | 2.4 | 51.1 |
|  |  |  |  | 2.6 | 0.1 | 0.4 | 8.4 | 2.0 | 0.1 | 63.1 | 9.9 | 2.2 | 2.5 | 49.1 |
|  |  |  |  | 3.3 | 0.1 | 0.6 | 8.5 | 1.9 | 0.1 | 60.0 | 10.1 | 3.1 | 2.3 | 51.2 |
|  |  |  |  | 2.5 | 0.1 | 0.4 | 9.2 | 2.0 | 0.1 | 62.3 | 9.6 | 2.0 | 2.5 | 48.1 |
|  |  |  |  | 6.8 | 0.1 | 1.1 | 12.1 | 1.6 | 0.2 | 51.7 | 11.2 | 3.6 | 2.2 | 55.2 |
|  |  |  |  | 2.8 | 0.1 | 0.4 | 8.4 | 2.0 | 0.1 | 63.5 | 10.0 | 2.2 | 2.2 | 51.4 |
|  |  |  |  | 3.4 | 0.1 | 0.6 | 8.2 | 1.7 | 0.2 | 60.1 | 9.9 | 3.3 | 2.4 | 50.3 |
| Jeong  et al.^15^ | Gobi Desert, East Asia | Sediment | < 20 µm | 7.5 | 0.1 | 1.2 | 3.1 | 3.7 | 0.3 | 61.8 | 18.0 | 2.5 | 1.8 | 64.5 |
|  |  |  |  | 4.6 | 0.1 | 0.5 | 23.3 | 2.0 | 0.2 | 51.3 | 13.7 | 2.7 | 1.5 | 65.6 |
|  |  |  |  | 6.6 | 0.1 | 1.1 | 2.2 | 2.8 | 0.2 | 66.3 | 16.1 | 2.5 | 2.1 | 60.5 |
|  |  |  |  | 6.8 | 0.1 | 1.0 | 5.0 | 3.0 | 0.3 | 62.3 | 16.6 | 3.2 | 1.8 | 64.8 |
|  |  |  |  | 7.2 | 0.2 | 0.8 | 5.9 | 3.2 | 0.4 | 60.3 | 16.7 | 3.6 | 1.8 | 63.7 |
|  |  |  |  | 7.2 | 0.1 | 1.0 | 4.2 | 3.0 | 0.3 | 61.7 | 17.4 | 3.4 | 1.8 | 65.9 |
|  |  |  |  | 8.1 | 0.2 | 1.0 | 5.2 | 3.2 | 0.3 | 58.3 | 17.5 | 4.1 | 2.3 | 61.3 |
|  |  |  |  | 6.3 | 0.1 | 1.0 | 7.3 | 2.7 | 0.3 | 62.0 | 15.8 | 3.0 | 1.6 | 65.5 |
|  |  |  |  | 6.2 | 0.1 | 1.0 | 9.9 | 2.6 | 0.2 | 59.7 | 15.6 | 2.9 | 1.8 | 64.2 |
|  |  |  |  | 6.2 | 0.1 | 0.9 | 10.1 | 2.6 | 0.3 | 59.1 | 15.8 | 3.1 | 1.9 | 64.1 |
|  |  |  |  | 7.1 | 0.1 | 0.8 | 7.8 | 3.3 | 0.3 | 56.9 | 18.1 | 4.2 | 1.3 | 69.4 |
|  |  |  |  | 7.3 | 0.2 | 0.8 | 7.5 | 3.2 | 0.3 | 57.6 | 17.8 | 3.9 | 1.4 | 68.5 |
|  |  |  |  | 6.9 | 0.1 | 0.9 | 7.8 | 3.2 | 0.3 | 58.9 | 16.9 | 3.6 | 1.6 | 66.1 |
|  |  |  |  | 7.0 | 0.1 | 1.0 | 8.2 | 2.9 | 0.3 | 58.7 | 16.4 | 3.5 | 1.8 | 64.3 |
|  |  |  |  | 6.8 | 0.1 | 1.0 | 6.2 | 2.8 | 0.3 | 61.3 | 16.4 | 3.2 | 2.0 | 63.4 |
|  |  |  |  | 7.1 | 0.1 | 0.9 | 8.7 | 2.9 | 0.3 | 57.1 | 17.6 | 3.8 | 1.4 | 69.0 |
|  |  |  |  | 7.6 | 0.1 | 0.9 | 5.8 | 3.3 | 0.3 | 59.3 | 17.7 | 3.6 | 1.5 | 67.8 |
|  |  |  |  | 6.3 | 0.1 | 1.1 | 6.3 | 2.4 | 0.3 | 63.3 | 15.5 | 2.5 | 2.3 | 60.4 |
|  |  |  |  | 6.3 | 0.1 | 0.8 | 20.0 | 2.8 | 0.2 | 50.1 | 15.1 | 3.3 | 1.3 | 67.6 |
|  |  |  |  | 6.9 | 0.1 | 0.8 | 6.0 | 3.2 | 0.3 | 60.8 | 17.3 | 3.3 | 1.4 | 68.6 |
|  |  |  |  | 6.7 | 0.1 | 0.9 | 10.6 | 3.0 | 0.4 | 56.9 | 16.0 | 3.8 | 1.6 | 65.0 |
|  |  |  |  | 7.0 | 0.2 | 0.8 | 10.6 | 3.1 | 0.3 | 54.7 | 16.2 | 4.5 | 2.7 | 56.8 |
|  |  |  |  | 5.8 | 0.1 | 0.9 | 11.6 | 2.3 | 0.2 | 59.3 | 14.6 | 3.0 | 2.3 | 59.3 |
|  |  |  |  | 6.2 | 0.1 | 1.0 | 9.2 | 2.9 | 0.3 | 59.9 | 14.7 | 3.3 | 2.4 | 57.3 |
|  |  |  |  | 5.8 | 0.1 | 0.8 | 12.5 | 2.6 | 0.2 | 57.4 | 14.9 | 3.3 | 2.2 | 59.8 |
|  |  |  |  | 6.5 | 0.1 | 0.9 | 8.8 | 2.7 | 0.3 | 59.7 | 15.5 | 3.4 | 2.1 | 61.4 |
|  |  |  |  | 6.5 | 0.1 | 1.0 | 6.1 | 2.5 | 0.3 | 62.7 | 15.7 | 3.1 | 2.1 | 61.6 |
|  |  |  |  | 6.3 | 0.1 | 0.8 | 18.1 | 2.7 | 0.2 | 51.8 | 15.4 | 3.2 | 1.4 | 66.9 |
|  |  |  |  | 6.4 | 0.1 | 0.8 | 11.2 | 2.4 | 0.2 | 57.4 | 15.5 | 3.7 | 2.2 | 61.4 |
|  |  |  |  | 6.4 | 0.1 | 0.8 | 11.3 | 2.3 | 0.2 | 57.6 | 15.4 | 3.5 | 2.2 | 61.5 |
|  |  |  |  | 7.6 | 0.2 | 0.8 | 7.4 | 3.2 | 0.3 | 53.8 | 18.7 | 4.7 | 3.5 | 55.8 |
|  |  |  |  | 6.6 | 0.1 | 0.9 | 10.4 | 2.9 | 0.3 | 57.3 | 16.3 | 3.8 | 1.5 | 66.4 |
|  |  |  |  | 7.7 | 0.2 | 1.1 | 8.4 | 3.2 | 0.4 | 57.1 | 17.6 | 2.8 | 1.6 | 66.8 |
|  |  |  |  | 5.6 | 0.1 | 0.7 | 5.6 | 2.3 | 0.1 | 61.0 | 19.2 | 3.1 | 2.3 | 65.5 |
| Pang et al.^16^ | Chinese Loess Plateau,  East Asia | Sediment | < 2 µm | 4.6 | – | – | 15.9 | 2.6 | – | 45.7 | 10.6 | 3.5 | 1.6 | 56.8 |
|  |  |  |  | 4.5 | – | – | 10.3 | 2.8 | – | 54.7 | 11.4 | 3.2 | 1.6 | 57.9 |
|  |  |  |  | 6.1 | – | – | 13.0 | 3.3 | – | 47.6 | 13.7 | 4.0 | 1.5 | 61.7 |
|  |  |  |  | 4.7 | – | – | 11.0 | 2.8 | – | 51.3 | 11.6 | 3.5 | 1.9 | 55.6 |
|  |  |  |  | 4.5 | – | – | 8.4 | 2.9 | – | 59.1 | 11.3 | 2.7 | 1.4 | 59.3 |
|  |  |  |  | 4.0 | – | – | 12.3 | 2.3 | – | 55.2 | 9.9 | 2.2 | 1.9 | 53.1 |
|  |  |  |  | 4.1 | – | – | 7.8 | 2.5 | – | 62.9 | 10.3 | 2.1 | 1.8 | 54.4 |
|  |  |  |  | 5.0 | – | – | 11.8 | 2.9 | – | 51.7 | 12.1 | 3.2 | 1.6 | 59.0 |
|  |  |  |  | 4.8 | – | – | 11.8 | 2.9 | – | 51.9 | 12.1 | 3.0 | 1.6 | 59.0 |
|  |  |  |  | 5.1 | – | – | 11.0 | 3.0 | – | 53.1 | 12.4 | 3.4 | 1.6 | 59.3 |
|  |  |  |  | 4.3 | – | – | 10.9 | 2.9 | – | 54.5 | 11.3 | 2.6 | 1.9 | 54.6 |
|  |  |  |  | 4.5 | – | – | 10.3 | 3.0 | – | 54.1 | 11.3 | 3.0 | 1.7 | 56.1 |
|  |  |  |  | 4.9 | – | – | 11.0 | 3.0 | – | 52.9 | 12.0 | 3.3 | 1.5 | 59.5 |
|  |  |  |  | 5.3 | – | – | 10.9 | 3.2 | – | 52.8 | 12.9 | 3.7 | 1.3 | 62.5 |
|  |  |  |  | 3.1 | – | – | 5.8 | 2.4 | – | 67.8 | 9.0 | 1.9 | 2.1 | 48.6 |
|  |  |  |  | 5.4 | – | – | 14.4 | 3.7 | – | 53.5 | 11.5 | 5.1 | 0.8 | 63.4 |
| Xie & Chi^17^ | Horqin Sandy Land,  Northern China,  East Asia | Sediment | < 63 µm | 3.6 | 0.1 | 0.7 | 6.0 | 2.6 | 0.1 | 64.3 | 11.2 | 1.6 | 2.3 | 51.6 |
|  |  |  |  | 2.8 | 0.1 | 0.8 | 2.5 | 3.0 | 0.1 | 74.2 | 11.1 | 0.7 | 2.7 | 47.5 |
|  |  |  |  | 3.5 | 0.1 | 0.6 | 3.0 | 2.9 | 0.1 | 71.4 | 11.3 | 0.8 | 2.7 | 48.3 |
|  |  |  |  | 3.1 | 0.1 | 0.7 | 1.3 | 3.1 | 0.1 | 73.9 | 11.8 | 0.6 | 2.7 | 53.9 |
|  |  |  |  | 2.7 | 0.0 | 0.7 | 1.3 | 3.1 | 0.1 | 75.2 | 11.7 | 0.5 | 2.8 | 53.3 |
|  |  |  |  | 2.7 | 0.0 | 0.6 | 1.3 | 3.0 | 0.1 | 74.9 | 11.7 | 0.6 | 2.8 | 53.2 |
|  |  |  |  | 3.2 | 0.1 | 0.8 | 1.3 | 3.1 | 0.1 | 73.1 | 11.8 | 0.7 | 2.6 | 54.0 |
|  |  |  |  | 4.1 | 0.1 | 0.9 | 2.3 | 2.7 | 0.1 | 71.1 | 11.8 | 0.8 | 2.5 | 50.9 |
|  |  |  |  | 3.1 | 0.1 | 0.8 | 1.4 | 2.8 | 0.0 | 73.7 | 11.9 | 0.7 | 2.7 | 54.2 |
|  |  |  |  | 2.6 | 0.1 | 0.8 | 1.9 | 3.0 | 0.0 | 74.1 | 11.5 | 0.6 | 2.8 | 50.7 |
|  |  |  | 11–30 µm | 3.9 | 0.1 | 1.0 | 1.4 | 2.6 | 0.1 | 69.6 | 13.1 | 1.1 | 2.3 | 58.8 |
|  |  |  |  | 3.5 | 0.1 | 0.9 | 3.1 | 2.6 | 0.1 | 70.1 | 12.0 | 1.3 | 2.7 | 51.0 |
|  |  |  |  | 3.3 | 0.1 | 0.7 | 8.8 | 2.4 | 0.1 | 58.3 | 11.1 | 2.3 | 2.0 | 54.4 |
|  |  |  |  | 2.8 | 0.1 | 0.8 | 3.3 | 2.7 | 0.1 | 70.3 | 11.5 | 1.2 | 2.6 | 50.3 |
|  |  |  |  | 3.1 | 0.1 | 0.7 | 1.6 | 2.8 | 0.1 | 70.1 | 12.6 | 1.0 | 2.5 | 55.6 |
|  |  |  |  | 2.5 | 0.1 | 0.8 | 1.5 | 2.8 | 0.1 | 73.4 | 11.9 | 0.8 | 2.8 | 53.6 |
|  |  |  |  | 1.8 | 0.0 | 0.6 | 1.1 | 2.5 | 0.1 | 63.8 | 10.1 | 0.6 | 2.6 | 53.0 |
|  |  |  |  | 2.4 | 0.0 | 0.6 | 1.5 | 2.8 | 0.1 | 74.3 | 11.9 | 0.8 | 2.8 | 53.4 |
|  |  |  |  | 3.2 | 0.1 | 1.0 | 3.0 | 2.6 | 0.1 | 68.8 | 12.2 | 1.1 | 2.5 | 52.7 |
|  |  |  |  | 3.2 | 0.1 | 0.9 | 1.7 | 2.7 | 0.1 | 72.7 | 12.4 | 1.0 | 2.6 | 54.8 |
|  |  |  | < 11 µm | 4.4 | 0.1 | 1.1 | 1.4 | 2.4 | 0.1 | 68.1 | 13.5 | 1.2 | 2.2 | 60.4 |
|  |  |  |  | 3.8 | 0.1 | 1.1 | 3.4 | 2.4 | 0.1 | 69.0 | 11.8 | 1.3 | 2.7 | 50.9 |
|  |  |  |  | 3.5 | 0.1 | 0.8 | 8.9 | 2.2 | 0.2 | 57.8 | 11.0 | 2.3 | 2.0 | 54.6 |
|  |  |  |  | 3.4 | 0.1 | 1.0 | 3.4 | 2.4 | 0.1 | 68.8 | 11.5 | 1.2 | 2.6 | 51.0 |
|  |  |  |  | 3.8 | 0.1 | 1.2 | 1.9 | 2.6 | 0.1 | 69.3 | 12.6 | 1.0 | 2.5 | 54.9 |
|  |  |  |  | 3.1 | 0.1 | 1.2 | 1.7 | 2.5 | 0.1 | 72.9 | 11.9 | 0.8 | 2.8 | 53.2 |
| Xie & Chi^17^ | Horqin Sandy Land,  Northern China,  East Asia | Sediment | < 11 µm | 2.4 | 0.1 | 1.0 | 1.6 | 2.6 | 0.1 | 73.9 | 12.1 | 0.8 | 2.9 | 53.4 |
|  |  |  |  | 2.8 | 0.1 | 0.9 | 1.7 | 2.5 | 0.1 | 73.2 | 11.8 | 0.8 | 2.8 | 53.3 |
|  |  |  |  | 3.7 | 0.1 | 1.2 | 3.4 | 2.4 | 0.1 | 66.8 | 12.2 | 1.2 | 2.4 | 54.1 |
|  |  |  |  | 3.7 | 0.1 | 1.1 | 1.7 | 2.5 | 0.1 | 70.5 | 12.5 | 1.0 | 2.5 | 55.8 |
|  | Hulum Buir Sandy Land, Northern China,  East Asia |  | < 63 µm | 3.2 | 0.1 | 0.7 | 1.5 | 3.1 | 0.1 | 71.5 | 13.0 | 0.8 | 3.0 | 54.3 |
|  |  |  |  | 5.3 | 0.1 | 0.8 | 1.5 | 2.9 | 0.1 | 65.3 | 12.8 | 0.7 | 2.4 | 56.7 |
|  |  |  |  | 2.7 | 0.0 | 0.7 | 1.4 | 3.1 | 0.1 | 74.1 | 12.4 | 0.6 | 3.1 | 53.0 |
|  |  |  |  | 2.6 | 0.1 | 0.7 | 1.5 | 3.1 | 0.1 | 74.1 | 12.4 | 0.5 | 3.1 | 52.7 |
|  |  |  |  | 5.9 | 0.1 | 1.9 | 4.7 | 2.5 | 0.1 | 62.6 | 12.9 | 1.6 | 2.8 | 52.4 |
|  |  |  |  | 3.2 | 0.1 | 1.0 | 1.6 | 3.2 | 0.1 | 73.4 | 12.4 | 0.6 | 3.2 | 51.7 |
|  |  |  |  | 5.1 | 0.1 | 0.9 | 1.7 | 2.9 | 0.1 | 66.8 | 12.7 | 0.8 | 2.7 | 54.7 |
|  |  |  |  | 6.7 | 0.2 | 2.4 | 1.9 | 3.1 | 0.1 | 71.8 | 11.6 | 0.6 | 2.7 | 50.8 |
|  |  |  |  | 3.2 | 0.1 | 1.1 | 1.6 | 3.2 | 0.1 | 74.6 | 11.6 | 0.5 | 2.9 | 51.1 |
|  |  |  |  | 5.5 | 0.1 | 1.2 | 1.5 | 2.9 | 0.1 | 67.6 | 12.6 | 0.8 | 2.7 | 54.9 |
|  |  |  |  | 4.5 | 0.1 | 0.7 | 1.4 | 2.9 | 0.1 | 67.6 | 13.0 | 0.8 | 2.6 | 56.8 |
|  |  |  |  | 5.2 | 0.1 | 1.7 | 1.7 | 3.0 | 0.1 | 69.4 | 11.7 | 0.6 | 2.8 | 51.8 |
|  |  |  |  | 3.4 | 0.1 | 1.2 | 1.6 | 3.1 | 0.1 | 72.8 | 12.2 | 0.6 | 2.9 | 52.7 |
| Pang et al.^16^ | Ordos Plateau, East Asia | Sediment | < 2 µm | 1.5 | – | – | 0.5 | 2.8 | – | 73.7 | 10.6 | 10.6 | 3.2 | 53.5 |
|  |  |  |  | 1.8 | – | – | 0.5 | 3.1 | – | 71.0 | 11.1 | 11.1 | 3.9 | 51.0 |
|  |  |  |  | 3.1 | – | – | 1.5 | 2.3 | – | 64.3 | 11.2 | 11.2 | 6.5 | 41.3 |
|  |  |  |  | 2.2 | – | – | 0.7 | 2.5 | – | 73.7 | 9.9 | 9.9 | 4.1 | 48.0 |
|  |  |  |  | 2.7 | – | – | 1.1 | 2.0 | – | 75.5 | 9.2 | 9.2 | 3.3 | 49.0 |
|  |  |  |  | 2.1 | – | – | 0.6 | 2.4 | – | 75.6 | 9.1 | 9.1 | 3.7 | 48.2 |
|  |  |  |  | 2.4 | – | – | 0.9 | 2.3 | – | 72.1 | 9.6 | 9.6 | 5.0 | 43.7 |
|  |  |  |  | 2.9 | – | – | 0.9 | 2.3 | – | 71.9 | 9.1 | 9.1 | 4.4 | 44.5 |
|  |  |  |  | 2.6 | – | – | 1.1 | 2.4 | – | 68.7 | 9.8 | 9.8 | 5.4 | 42.1 |
|  |  |  |  | 3.2 | – | – | 0.9 | 2.3 | – | 72.8 | 9.4 | 9.4 | 4.5 | 44.9 |
|  |  |  |  | 2.3 | – | – | 0.8 | 2.4 | – | 72.9 | 9.3 | 9.3 | 4.4 | 45.2 |
|  |  |  |  | 3.1 | – | – | 1.9 | 2.1 | – | 67.1 | 9.8 | 9.8 | 5.6 | 39.6 |
|  |  |  |  | 2.0 | – | – | 0.6 | 1.8 | – | 80.3 | 8.2 | 8.2 | 2.4 | 54.0 |
|  |  |  |  | 2.1 | – | – | 1.7 | 1.9 | – | 67.7 | 9.1 | 9.1 | 7.0 | 35.3 |
|  |  |  |  | 3.0 | – | – | 1.6 | 2.3 | – | 63.0 | 10.3 | 10.3 | 6.9 | 38.1 |
|  |  |  |  | 2.3 | – | – | 0.9 | 2.3 | – | 71.0 | 9.5 | 9.5 | 5.3 | 42.5 |
|  |  |  |  | 2.4 | – | – | 0.9 | 1.8 | – | 71.9 | 8.3 | 8.3 | 5.2 | 40.6 |
|  |  |  |  | 2.4 | – | – | 0.8 | 2.1 | – | 75.5 | 9.0 | 9.0 | 3.5 | 48.7 |
|  |  |  |  | 1.4 | – | – | 0.7 | 1.9 | – | 63.1 | 8.8 | 8.8 | 7.8 | 35.3 |
|  |  |  |  | 2.9 | – | – | 1.0 | 2.1 | – | 73.3 | 9.8 | 9.8 | 3.6 | 49.5 |
|  |  |  |  | 2.4 | – | – | 0.9 | 2.0 | – | 75.8 | 8.6 | 8.6 | 3.7 | 46.5 |
|  |  |  |  | 2.6 | – | – | 1.1 | 2.1 | – | 72.9 | 9.6 | 9.6 | 4.2 | 46.2 |
|  |  |  |  | 2.2 | – | – | 0.9 | 2.1 | – | 75.1 | 9.4 | 9.4 | 3.5 | 49.3 |
| Pang et al.^16^ | Ordos Plateau, East Asia | Sediment | < 2 µm | 1.3 | – | – | 0.3 | 1.9 | – | 77.2 | 7.5 | 7.5 | 3.7 | 46.3 |
|  |  |  |  | 2.0 | – | – | 0.6 | 2.7 | – | 73.8 | 10.2 | 10.2 | 3.5 | 51.1 |
|  |  |  |  | 2.3 | – | – | 0.8 | 2.3 | – | 74.9 | 9.6 | 9.6 | 3.5 | 49.7 |
|  |  |  |  | 2.1 | – | – | 0.9 | 2.3 | – | 71.2 | 9.3 | 9.3 | 4.5 | 44.7 |
|  |  |  |  | 1.4 | – | – | 0.6 | 2.1 | – | 77.3 | 8.5 | 8.5 | 3.3 | 49.2 |
|  |  |  |  | 1.8 | – | – | 0.6 | 1.9 | – | 80.0 | 8.3 | 8.3 | 2.1 | 55.7 |
|  |  |  |  | 2.0 | – | – | 0.6 | 2.0 | – | 78.6 | 8.9 | 8.9 | 2.2 | 56.4 |
|  |  |  |  | 1.5 | – | – | 0.7 | 2.0 | – | 72.0 | 9.0 | 9.0 | 4.5 | 45.4 |
|  |  |  |  | 2.3 | – | – | 0.6 | 2.0 | – | 74.2 | 8.3 | 8.3 | 3.8 | 46.6 |
|  |  |  |  | 3.6 | – | – | 2.2 | 2.1 | – | 60.8 | 10.7 | 10.7 | 8.1 | 35.3 |
|  |  |  |  | 3.3 | – | – | 1.9 | 2.3 | – | 63.2 | 10.2 | 10.2 | 7.3 | 36.2 |
|  |  |  |  | 2.2 | – | – | 0.7 | 2.2 | – | 77.1 | 8.7 | 8.7 | 3.0 | 50.3 |
|  |  |  |  | 2.6 | – | – | 1.1 | 2.3 | – | 71.6 | 9.8 | 9.8 | 3.7 | 48.1 |
|  |  |  |  | 3.6 | – | – | 1.9 | 2.5 | – | 61.7 | 9.9 | 9.9 | 7.7 | 34.5 |
|  |  |  |  | 1.5 | – | – | 0.6 | 2.7 | – | 75.5 | 9.2 | 9.2 | 2.3 | 54.1 |
|  |  |  |  | 1.6 | – | – | 0.6 | 2.8 | – | 74.2 | 9.4 | 9.4 | 2.4 | 53.8 |
|  |  |  |  | 2.0 | – | – | 0.9 | 2.2 | – | 72.7 | 9.3 | 9.3 | 3.4 | 49.2 |
|  |  |  |  | 1.7 | – | – | 0.8 | 2.4 | – | 71.9 | 8.6 | 8.6 | 4.3 | 43.6 |
|  |  |  |  | 1.7 | – | – | 0.8 | 2.3 | – | 70.0 | 8.0 | 8.0 | 4.4 | 41.7 |
|  |  |  |  | 1.6 | – | – | 0.7 | 2.1 | – | 75.6 | 8.2 | 8.2 | 2.8 | 50.1 |
|  |  |  |  | 2.1 | – | – | 0.6 | 1.9 | – | 81.2 | 9.2 | 9.2 | 1.6 | 61.4 |
|  |  |  |  | 2.0 | – | – | 0.6 | 3.3 | – | 67.8 | 12.1 | 12.1 | 4.8 | 49.1 |
|  |  |  |  | 3.0 | – | – | 2.3 | 2.4 | – | 61.8 | 9.5 | 9.5 | 8.2 | 31.9 |
|  |  |  |  | 3.7 | – | – | 2.3 | 2.4 | – | 59.4 | 10.3 | 10.3 | 8.5 | 33.2 |
| Bücher & Lucas^18^ | Pyrenees, Europe (Northwestern Sahara) | Dust | 4–13 µm  (median) | 6.1 | 0.1 | – | 10.2 | 2.2 | 0.1 | 54.8 | 16.3 | 2.9 | 1.0 | 74.5 |
| Bücher^19^ |  |  | 3–12 µm  (mean) | 4.0 | – | 2.0 | 8.2 | 0.5 | – | 52.0 | 16.4 | 2.0 | 0.6 | 86.7 |
|  |  |  |  | 6.4 | – | 1.0 | 11.0 | 2.8 | – | 56.0 | 15.0 | 3.2 | 1.2 | 68.2 |
|  |  |  |  | 7.1 | 0.1 | 1.0 | 12.1 | 2.4 | – | 54.6 | 17.7 | 3.6 | 0.4 | 81.9 |
| Orange  et al.^20^ | Senegal, North Africa | Dust | Almost  < 50 µm | 5.4 | 0.1 | 0.9 | 2.2 | 1.8 | 0.2 | 73.8 | 11.6 | 1.5 | 0.8 | 72.4 |
|  |  |  |  | 5.2 | 0.1 | 0.9 | 1.0 | 1.0 | 0.2 | 76.5 | 10.1 | 1.5 | 0.1 | 86.7 |
|  |  |  |  | 5.2 | 0.1 | 0.9 | 0.9 | 1.1 | 0.1 | 76.8 | 10.3 | 1.3 | 0.1 | 87.1 |
| Herrmann  et al.^21^ | Lanzarote, Spain  (Western Sahara) |  | < 63 µm | 4.7 | – | – | – | 1.5 | – | 52.3 | 11.7 | 4.6 | 1.4 | 74.9 |
| Criado & Dorta^22^ | Tenerife, Spain  (Western Sahara) | Dust | Generally  < 63 µm | 5.0 | 0.1 | 0.9 | 9.6 | 2.0 | 0.4 | 50.4 | 11.8 | 3.5 | 0.9 | 69.9 |
| Linke et al.^23^ | Morocco, North Africa | Soil | < 20 µm | 3.6 | – | 0.8 | 11.5 | 2.0 | – | 70.2 | 8.4 | 2.3 | 0.5 | 68.1 |
| Castillo  et al.^24^ | Morocco, North Africa | Soil | 0.3–1.5 µm | 6.4 | 0.1 | 1.0 | 12.1 | 2.4 | 0.3 | 54.0 | 12.5 | 3.5 | 0.9 | 71.4 |
|  |  |  | 1.5–3.0 µm | 6.6 | 0.1 | 0.8 | 6.2 | 1.9 | 0.3 | 54.4 | 12.6 | 3.7 | 0.9 | 70.7 |
|  |  |  | 3.0–12 µm | 5.5 | 0.1 | 0.7 | 3.0 | 2.1 | 0.3 | 50.0 | 11.6 | 3.8 | 0.9 | 68.9 |
| Fu et al.^25^ | Carpathian Basin,  Europe | Loess/  Paleosol | < 2 µm | 5.4 | 0.1 | 1.0 | 1.8 | 2.4 | 0.2 | 71.1 | 13.9 | 2.6 | 1.6 | 64.1 |
|  |  |  |  | 5.7 | 0.1 | 1.1 | 1.5 | 2.5 | 0.2 | 69.8 | 15.0 | 2.3 | 1.9 | 63.7 |
|  |  |  |  | 5.4 | 0.1 | 1.0 | 1.5 | 2.5 | 0.2 | 71.0 | 14.4 | 2.3 | 1.7 | 63.9 |
|  |  |  |  | 5.5 | 0.1 | 1.0 | 1.5 | 2.5 | 0.3 | 71.4 | 14.1 | 2.1 | 1.5 | 64.2 |
|  |  |  |  | 5.4 | 0.1 | 1.0 | 1.8 | 2.4 | 0.2 | 70.7 | 14.1 | 2.6 | 1.7 | 63.4 |
|  |  |  |  | 5.9 | 0.1 | 1.0 | 1.5 | 2.5 | 0.2 | 69.2 | 15.5 | 2.3 | 1.9 | 64.7 |
|  |  |  |  | 5.6 | 0.1 | 1.0 | 1.6 | 2.6 | 0.2 | 69.8 | 15.1 | 2.3 | 1.8 | 63.6 |
|  |  |  |  | 5.7 | 0.1 | 1.1 | 1.7 | 2.7 | 0.2 | 68.6 | 15.5 | 2.4 | 2.0 | 62.6 |
|  |  |  |  | 5.5 | 0.1 | 1.0 | 2.0 | 2.6 | 0.2 | 68.6 | 15.4 | 2.5 | 2.1 | 60.8 |
|  |  |  |  | 5.5 | 0.1 | 1.0 | 1.5 | 2.3 | 0.2 | 71.0 | 14.7 | 2.2 | 1.5 | 65.5 |
|  |  |  |  | 5.5 | 0.1 | 1.0 | 1.3 | 2.3 | 0.2 | 71.8 | 14.5 | 2.1 | 1.4 | 67.3 |
|  |  |  |  | 5.4 | 0.1 | 1.0 | 1.8 | 2.4 | 0.2 | 71.1 | 13.9 | 2.6 | 1.6 | 64.1 |
|  |  |  |  | 5.7 | 0.1 | 1.1 | 1.5 | 2.5 | 0.2 | 69.8 | 15.0 | 2.3 | 1.9 | 63.7 |
| Muhs et al.^26^ | Nebraska, U.S. | Loess | Bulk | 3.7 | 0.1 | 0.6 | 1.3 | 2.8 | 0.2 | 71.6 | 12.8 | 1.3 | 1.3 | 62.8 |
|  |  |  |  | 3.7 | 0.1 | 0.5 | 3.6 | 2.8 | 0.2 | 67.2 | 11.8 | 1.9 | 1.3 | 62.3 |
|  |  |  |  | 3.9 | 0.1 | 0.5 | 1.7 | 2.8 | 0.1 | 70.8 | 12.5 | 1.4 | 1.3 | 62.9 |
|  |  |  |  | 3.7 | 0.1 | 0.5 | 3.9 | 2.7 | 0.2 | 67.6 | 11.8 | 1.9 | 1.3 | 62.3 |
|  |  |  |  | 3.5 | 0.1 | 0.6 | 2.9 | 2.6 | 0.1 | 69.4 | 11.8 | 1.7 | 1.4 | 61.7 |
|  |  |  |  | 4.0 | 0.1 | 0.6 | 1.6 | 2.7 | 0.2 | 71.6 | 12.7 | 1.4 | 1.3 | 63.7 |
|  |  |  |  | 4.0 | 0.1 | 0.6 | 1.8 | 2.7 | 0.2 | 70.8 | 12.6 | 1.4 | 1.3 | 63.6 |
|  |  |  |  | 3.5 | 0.1 | 0.5 | 1.8 | 2.9 | 0.1 | 71.5 | 11.9 | 1.3 | 1.4 | 60.5 |
|  |  |  |  | 3.4 | 0.1 | 0.5 | 2.9 | 2.8 | 0.2 | 69.5 | 11.5 | 1.6 | 1.4 | 59.6 |
|  |  |  |  | 3.5 | 0.1 | 0.5 | 3.8 | 2.7 | 0.1 | 68.2 | 11.6 | 1.7 | 1.3 | 61.6 |
|  |  |  |  | 3.4 | 0.1 | 0.5 | 1.9 | 2.8 | 0.1 | 72.6 | 12.1 | 1.3 | 1.4 | 60.9 |
|  |  |  |  | 3.5 | 0.1 | 0.5 | 1.7 | 2.9 | 0.2 | 72.4 | 12.3 | 1.3 | 1.4 | 61.1 |
|  |  |  |  | 3.2 | 0.0 | 0.5 | 3.2 | 2.8 | 0.1 | 70.0 | 11.8 | 1.7 | 1.4 | 61.1 |
|  |  |  |  | 3.5 | 0.1 | 0.5 | 2.7 | 2.9 | 0.1 | 69.3 | 11.9 | 1.8 | 1.3 | 61.4 |
|  |  |  |  | 3.4 | 0.1 | 0.5 | 2.9 | 2.7 | 0.1 | 70.1 | 11.7 | 1.9 | 1.3 | 61.9 |
|  |  |  |  | 3.3 | 0.1 | 0.5 | 2.9 | 2.8 | 0.1 | 69.6 | 11.6 | 1.6 | 1.5 | 59.7 |
|  |  |  |  | 3.2 | 0.1 | 0.5 | 3.2 | 3.0 | 0.1 | 68.4 | 11.4 | 1.7 | 1.4 | 59.4 |
|  |  |  |  | 3.0 | 0.0 | 0.5 | 3.0 | 3.0 | 0.1 | 70.3 | 11.4 | 1.5 | 1.5 | 58.2 |
|  |  |  |  | 3.1 | 0.0 | 0.5 | 2.8 | 3.0 | 0.1 | 70.5 | 11.5 | 1.5 | 1.4 | 59.2 |
|  |  |  |  | 3.0 | 0.0 | 0.5 | 2.7 | 3.0 | 0.1 | 70.4 | 11.5 | 1.6 | 1.4 | 59.1 |
|  |  |  |  | 3.2 | 0.1 | 0.5 | 3.7 | 2.8 | 0.1 | 69.4 | 11.6 | 1.7 | 1.3 | 60.9 |
|  |  |  |  | 3.1 | 0.0 | 0.5 | 1.9 | 3.0 | 0.1 | 72.6 | 11.7 | 1.2 | 1.8 | 56.4 |
|  |  |  |  | 3.0 | 0.0 | 0.5 | 2.1 | 3.0 | 0.1 | 72.5 | 11.7 | 1.3 | 1.5 | 59.0 |
|  |  |  |  | 3.0 | 0.0 | 0.5 | 2.7 | 2.8 | 0.1 | 71.2 | 11.6 | 1.3 | 1.5 | 59.3 |
|  |  |  |  | 2.9 | 0.0 | 0.5 | 3.2 | 2.9 | 0.1 | 71.9 | 11.6 | 1.3 | 1.6 | 58.0 |
|  |  |  |  | 2.8 | 0.0 | 0.5 | 2.7 | 2.9 | 0.1 | 73.5 | 11.7 | 1.2 | 1.7 | 57.6 |
| Muhs et al.^26^ | Nebraska, U.S. | Loess | Bulk | 2.9 | 0.0 | 0.5 | 3.1 | 2.9 | 0.1 | 71.1 | 11.5 | 1.4 | 1.5 | 58.7 |
|  |  |  |  | 3.2 | 0.1 | 0.6 | 3.5 | 2.8 | 0.1 | 70.5 | 11.3 | 1.4 | 1.5 | 58.5 |
|  |  |  |  | 1.8 | 0.1 | 0.3 | 1.6 | 2.3 | 0.1 | 81.3 | 8.5 | 0.6 | 1.3 | 55.1 |
|  |  |  |  | 2.7 | 0.0 | 0.5 | 2.8 | 2.8 | 0.1 | 71.9 | 11.1 | 1.3 | 1.5 | 57.9 |
|  |  |  |  | 2.6 | 0.0 | 0.4 | 3.0 | 2.9 | 0.1 | 71.8 | 11.1 | 1.2 | 1.6 | 57.3 |
|  |  |  |  | 2.5 | 0.0 | 0.4 | 2.9 | 2.9 | 0.1 | 73.8 | 11.1 | 1.1 | 1.7 | 56.1 |
|  |  |  |  | 3.5 | 0.1 | 0.6 | 1.6 | 2.6 | 0.1 | 69.6 | 12.2 | 1.3 | 1.5 | 61.3 |
|  |  |  |  | 2.7 | 0.0 | 0.5 | 2.2 | 2.8 | 0.1 | 71.4 | 11.3 | 1.1 | 1.6 | 57.3 |
|  |  |  |  | 2.4 | 0.0 | 0.4 | 2.7 | 2.8 | 0.1 | 74.1 | 11.3 | 1.1 | 1.8 | 56.2 |
|  |  |  |  | 2.5 | 0.0 | 0.4 | 3.8 | 2.8 | 0.1 | 72.5 | 10.7 | 1.3 | 1.6 | 56.6 |
|  |  |  |  | 3.5 | 0.0 | 0.6 | 1.5 | 2.9 | 0.2 | 72.6 | 12.4 | 1.3 | 1.4 | 60.4 |
|  |  |  |  | 3.3 | 0.1 | 0.5 | 6.2 | 2.5 | 0.1 | 65.5 | 11.3 | 1.8 | 1.2 | 62.8 |
|  |  |  |  | 3.4 | 0.1 | 0.5 | 4.7 | 2.7 | 0.1 | 67.1 | 11.7 | 1.8 | 1.2 | 62.7 |
|  |  |  |  | 3.4 | 0.1 | 0.6 | 2.8 | 2.8 | 0.1 | 70.3 | 12.0 | 1.6 | 1.4 | 61.6 |
|  |  |  |  | 3.5 | 0.1 | 0.6 | 1.7 | 2.9 | 0.2 | 72.2 | 12.3 | 1.4 | 1.3 | 62.0 |
|  |  |  |  | 3.3 | 0.1 | 0.5 | 3.4 | 3.0 | 0.1 | 68.8 | 11.6 | 1.6 | 1.4 | 59.8 |
|  |  |  |  | 3.3 | 0.1 | 0.6 | 3.2 | 2.8 | 0.1 | 69.6 | 11.9 | 1.7 | 1.4 | 60.8 |
|  |  |  |  | 3.2 | 0.1 | 0.5 | 3.1 | 2.8 | 0.1 | 70.7 | 11.5 | 1.6 | 1.4 | 60.5 |
|  |  |  |  | 3.5 | 0.1 | 0.5 | 4.9 | 2.7 | 0.1 | 65.8 | 11.4 | 1.8 | 1.2 | 62.2 |
|  |  |  |  | 4.3 | 0.1 | 0.6 | 2.2 | 2.6 | 0.2 | 68.8 | 12.5 | 2.1 | 1.2 | 64.7 |
|  |  |  |  | 3.5 | 0.1 | 0.6 | 3.9 | 2.6 | 0.2 | 66.3 | 11.9 | 2.0 | 1.2 | 64.0 |
|  |  |  |  | 3.7 | 0.1 | 0.6 | 3.8 | 2.5 | 0.2 | 66.9 | 11.8 | 2.0 | 1.3 | 63.3 |
|  |  |  |  | 3.9 | 0.1 | 0.6 | 3.5 | 2.5 | 0.2 | 67.9 | 11.7 | 2.1 | 1.2 | 63.7 |
|  |  |  |  | 3.4 | 0.0 | 0.6 | 3.1 | 2.6 | 0.2 | 67.0 | 12.1 | 2.2 | 1.2 | 63.8 |
|  |  |  |  | 4.4 | 0.1 | 0.6 | 3.2 | 2.6 | 0.2 | 66.4 | 12.1 | 2.1 | 1.2 | 64.5 |
|  |  |  |  | 3.9 | 0.2 | 0.5 | 4.7 | 2.4 | 0.2 | 67.5 | 10.6 | 2.1 | 1.3 | 61.4 |
|  |  |  |  | 3.8 | 0.1 | 0.5 | 5.8 | 2.3 | 0.2 | 65.2 | 10.7 | 2.2 | 1.2 | 62.2 |
|  |  |  |  | 4.0 | 0.1 | 0.5 | 5.5 | 2.4 | 0.2 | 65.4 | 10.9 | 2.1 | 1.2 | 62.3 |
|  |  |  |  | 3.6 | 0.1 | 0.5 | 5.8 | 2.5 | 0.2 | 64.6 | 10.5 | 1.9 | 1.3 | 60.7 |
|  |  |  |  | 4.0 | 0.2 | 0.5 | 5.7 | 2.3 | 0.2 | 66.0 | 10.6 | 2.0 | 1.2 | 62.2 |
|  |  |  |  | 3.7 | 0.1 | 0.5 | 5.7 | 2.4 | 0.2 | 64.4 | 10.4 | 2.5 | 1.2 | 61.2 |
|  |  |  |  | 4.1 | 0.1 | 0.6 | 3.0 | 2.6 | 0.2 | 67.0 | 11.6 | 2.3 | 1.2 | 63.3 |
|  |  |  |  | 2.9 | 0.1 | 0.5 | 3.4 | 2.8 | 0.1 | 71.1 | 10.8 | 1.5 | 1.5 | 57.5 |
|  |  |  |  | 3.0 | 0.1 | 0.5 | 6.3 | 2.4 | 0.1 | 67.5 | 10.6 | 1.3 | 1.4 | 59.6 |
|  |  |  |  | 3.7 | 0.1 | 0.6 | 3.1 | 2.6 | 0.2 | 67.8 | 11.8 | 2.0 | 1.3 | 63.0 |
|  |  |  |  | 3.0 | 0.1 | 0.5 | 2.4 | 2.8 | 0.1 | 73.4 | 11.1 | 1.1 | 1.6 | 57.7 |
|  |  |  |  | 3.1 | 0.1 | 0.5 | 3.1 | 2.7 | 0.1 | 71.9 | 11.1 | 1.6 | 1.5 | 59.0 |
|  |  |  |  | 3.6 | 0.1 | 0.5 | 2.9 | 2.8 | 0.2 | 70.2 | 11.8 | 1.3 | 1.4 | 60.9 |
|  |  |  |  | 3.7 | 0.1 | 0.6 | 1.6 | 2.8 | 0.2 | 70.7 | 12.2 | 1.4 | 1.3 | 62.5 |
|  |  |  |  | 3.4 | 0.1 | 0.5 | 4.1 | 2.8 | 0.1 | 67.0 | 11.5 | 1.7 | 1.3 | 61.3 |
|  |  |  |  | 3.4 | 0.1 | 0.6 | 1.8 | 2.9 | 0.2 | 72.7 | 12.0 | 1.3 | 1.4 | 60.6 |
|  |  |  |  | 3.2 | 0.1 | 0.5 | 2.4 | 2.9 | 0.1 | 71.3 | 11.6 | 1.5 | 1.4 | 60.0 |
|  |  |  |  | 3.2 | 0.1 | 0.5 | 2.2 | 3.0 | 0.1 | 71.0 | 12.0 | 1.5 | 1.4 | 60.6 |
| Muhs et al.^26^ | Nebraska, U.S. | Loess | Bulk | 4.1 | 0.1 | 0.6 | 1.4 | 2.7 | 0.2 | 71.5 | 13.0 | 1.6 | 1.3 | 63.1 |
|  |  |  |  | 4.1 | 0.1 | 0.6 | 1.6 | 2.7 | 0.2 | 71.5 | 13.0 | 1.5 | 1.3 | 64.8 |
|  |  |  |  | 4.2 | 0.1 | 0.6 | 1.8 | 2.6 | 0.2 | 70.7 | 13.2 | 1.6 | 1.4 | 64.4 |
| Zech et al.^27^ | Verkhoyansk Mountains, Siberia | Loess/  Paleosol | < 2 mm | 3.7 | 0.1 | 0.8 | 0.7 | 2.0 | 0.1 | 71.8 | 12.5 | 1.0 | 2.8 | 61.1 |
|  |  |  |  | 5.1 | 0.1 | 0.8 | 0.8 | 2.6 | 0.1 | 66.1 | 14.8 | 1.5 | 2.4 | 64.5 |
|  |  |  |  | 4.6 | 0.1 | 0.7 | 2.3 | 2.5 | 0.1 | 66.1 | 14.0 | 1.5 | 2.4 | 56.4 |
|  |  |  |  | 4.6 | 0.1 | 0.7 | 1.8 | 2.6 | 0.1 | 66.1 | 14.0 | 1.5 | 2.4 | 58.2 |
|  |  |  |  | 4.7 | 0.1 | 0.7 | 1.6 | 2.6 | 0.1 | 66.9 | 14.2 | 1.6 | 2.5 | 59.1 |
|  |  |  |  | 4.4 | 0.1 | 0.7 | 1.0 | 2.4 | 0.1 | 68.6 | 13.4 | 1.3 | 2.6 | 60.9 |
|  |  |  |  | 4.4 | 0.1 | 0.7 | 1.3 | 2.4 | 0.1 | 69.0 | 13.2 | 1.4 | 2.6 | 58.9 |
|  |  |  |  | 4.4 | 0.1 | 0.7 | 1.3 | 2.4 | 0.1 | 67.8 | 13.2 | 1.4 | 2.5 | 59.0 |
|  |  |  |  | 4.4 | 0.1 | 0.7 | 2.4 | 2.3 | 0.1 | 66.8 | 13.0 | 1.5 | 2.5 | 54.2 |
|  |  |  |  | 4.2 | 0.1 | 0.7 | 1.3 | 2.4 | 0.1 | 66.5 | 13.3 | 1.5 | 2.5 | 59.4 |
|  |  |  |  | 4.3 | 0.1 | 0.7 | 1.0 | 2.5 | 0.1 | 67.1 | 13.6 | 1.5 | 2.5 | 61.2 |
|  |  |  |  | 4.3 | 0.1 | 0.7 | 3.8 | 2.3 | 0.1 | 64.1 | 13.1 | 1.7 | 2.3 | 56.1 |
|  |  |  |  | 4.4 | 0.1 | 0.7 | 2.8 | 2.4 | 0.1 | 64.9 | 13.4 | 1.6 | 2.4 | 55.9 |
|  |  |  |  | 4.7 | 0.1 | 0.7 | 2.5 | 2.5 | 0.1 | 64.4 | 13.9 | 1.8 | 2.4 | 56.9 |
|  |  |  |  | 4.8 | 0.1 | 0.7 | 1.9 | 2.6 | 0.1 | 64.8 | 14.1 | 1.7 | 2.4 | 58.0 |
|  |  |  |  | 4.2 | 0.1 | 0.7 | 2.4 | 2.5 | 0.1 | 65.3 | 13.5 | 1.7 | 2.4 | 55.8 |
|  |  |  |  | 4.3 | 0.1 | 0.7 | 3.0 | 2.3 | 0.1 | 66.0 | 13.2 | 1.8 | 2.5 | 55.2 |
|  |  |  |  | 4.8 | 0.1 | 0.8 | 1.3 | 2.6 | 0.1 | 66.1 | 14.5 | 1.6 | 2.4 | 61.2 |
|  |  |  |  | 4.4 | 0.1 | 0.7 | 1.3 | 2.6 | 0.1 | 66.2 | 14.2 | 1.5 | 2.5 | 60.4 |
|  |  |  |  | 4.6 | 0.1 | 0.7 | 1.5 | 2.6 | 0.1 | 66.2 | 14.1 | 1.5 | 2.5 | 59.4 |
|  |  |  |  | 4.7 | 0.1 | 0.8 | 1.2 | 2.6 | 0.1 | 65.6 | 14.3 | 1.5 | 2.4 | 61.3 |
|  |  |  |  | 4.4 | 0.1 | 0.7 | 1.1 | 2.6 | 0.2 | 66.7 | 14.0 | 1.5 | 2.5 | 61.1 |
|  |  |  |  | 4.4 | 0.1 | 0.7 | 1.3 | 2.6 | 0.2 | 66.1 | 14.1 | 1.5 | 2.5 | 60.2 |
|  |  |  |  | 4.8 | 0.1 | 0.7 | 1.9 | 2.6 | 0.2 | 65.1 | 13.8 | 1.6 | 2.5 | 57.2 |
|  |  |  |  | 4.4 | 0.1 | 0.7 | 1.4 | 2.7 | 0.2 | 66.2 | 14.1 | 1.5 | 2.5 | 59.6 |
|  |  |  |  | 4.3 | 0.1 | 0.7 | 1.2 | 2.7 | 0.1 | 65.9 | 14.1 | 1.5 | 2.5 | 60.6 |
|  |  |  |  | 4.5 | 0.1 | 0.8 | 1.2 | 2.7 | 0.2 | 66.4 | 14.2 | 1.6 | 2.5 | 60.5 |
|  |  |  |  | 4.6 | 0.1 | 0.7 | 1.5 | 2.6 | 0.2 | 65.3 | 14.0 | 1.5 | 2.5 | 58.8 |
|  |  |  |  | 4.6 | 0.1 | 0.7 | 1.6 | 2.7 | 0.2 | 65.5 | 14.0 | 1.6 | 2.5 | 58.8 |
|  |  |  |  | 4.4 | 0.1 | 0.7 | 1.5 | 2.6 | 0.2 | 65.2 | 13.8 | 1.6 | 2.5 | 58.7 |
|  |  |  |  | 4.4 | 0.1 | 0.7 | 1.5 | 2.7 | 0.2 | 65.6 | 14.0 | 1.6 | 2.5 | 58.8 |
|  |  |  |  | 5.6 | 0.1 | 0.8 | 0.9 | 2.6 | 0.1 | 64.5 | 15.5 | 1.6 | 2.1 | 65.8 |
|  |  |  |  | 5.3 | 0.1 | 0.8 | 1.0 | 2.6 | 0.1 | 65.0 | 15.7 | 1.6 | 2.1 | 66.1 |
|  |  |  |  | 5.8 | 0.1 | 0.8 | 1.0 | 2.6 | 0.1 | 64.2 | 15.5 | 1.6 | 2.1 | 65.8 |
|  |  |  |  | 5.1 | 0.2 | 0.8 | 1.5 | 2.5 | 0.2 | 64.4 | 14.1 | 1.6 | 2.4 | 60.2 |
|  |  |  |  | 5.3 | 0.1 | 0.8 | 1.1 | 2.6 | 0.1 | 65.5 | 15.0 | 1.5 | 2.3 | 63.7 |
|  |  |  |  | 5.3 | 0.1 | 0.8 | 1.1 | 2.6 | 0.1 | 65.0 | 14.9 | 1.5 | 2.3 | 63.7 |
|  |  |  |  | 5.3 | 0.1 | 0.8 | 1.5 | 2.6 | 0.1 | 65.3 | 14.9 | 1.7 | 2.3 | 61.4 |
|  |  |  |  | 5.3 | 0.1 | 0.8 | 1.4 | 2.6 | 0.1 | 64.7 | 14.8 | 1.7 | 2.3 | 61.8 |
|  |  |  |  | 5.2 | 0.1 | 0.8 | 1.9 | 2.6 | 0.1 | 63.8 | 14.7 | 1.8 | 2.3 | 59.5 |
| Zech et al.^27^ | Verkhoyansk Mountains, Siberia | Loess/  Paleosol | < 2 mm | 5.3 | 0.1 | 0.8 | 1.8 | 2.6 | 0.1 | 64.4 | 14.8 | 1.8 | 2.3 | 60.2 |
|  |  |  |  | 5.1 | 0.1 | 0.8 | 1.6 | 2.6 | 0.1 | 64.4 | 14.6 | 1.7 | 2.3 | 60.9 |
|  |  |  |  | 5.2 | 0.1 | 0.8 | 1.8 | 2.6 | 0.1 | 64.5 | 14.7 | 1.7 | 2.3 | 59.9 |
|  |  |  |  | 5.2 | 0.1 | 0.8 | 1.5 | 2.5 | 0.1 | 64.5 | 14.5 | 1.7 | 2.3 | 60.8 |
|  |  |  |  | 4.3 | 0.1 | 0.7 | 1.2 | 2.7 | 0.2 | 66.9 | 14.1 | 1.6 | 2.5 | 60.4 |
|  |  |  |  | 5.0 | 0.1 | 0.8 | 1.6 | 2.5 | 0.2 | 64.8 | 14.0 | 1.6 | 2.4 | 59.7 |
|  |  |  |  | 5.1 | 0.1 | 0.8 | 1.7 | 2.5 | 0.2 | 63.9 | 14.2 | 1.6 | 2.3 | 59.7 |
|  |  |  |  | 4.9 | 0.1 | 0.8 | 1.7 | 2.5 | 0.2 | 65.7 | 14.2 | 1.6 | 2.4 | 59.1 |
|  |  |  |  | 5.2 | 0.1 | 0.8 | 1.8 | 2.4 | 0.2 | 65.2 | 14.0 | 1.5 | 2.3 | 59.0 |
|  |  |  |  | 5.0 | 0.2 | 0.8 | 1.6 | 2.4 | 0.2 | 65.2 | 14.0 | 1.5 | 2.4 | 59.5 |
|  |  |  |  | 6.6 | 0.4 | 0.8 | 2.3 | 2.4 | 0.2 | 61.8 | 13.6 | 1.5 | 2.2 | 58.1 |
|  |  |  |  | 16.4 | 2.1 | 0.6 | 6.2 | 1.8 | 0.3 | 45.3 | 10.4 | 1.3 | 1.6 | 59.5 |
|  |  |  |  | 4.8 | 0.1 | 0.8 | 1.9 | 2.6 | 0.1 | 65.2 | 14.6 | 1.6 | 2.3 | 59.3 |
|  |  |  |  | 5.4 | 0.1 | 0.8 | 2.4 | 2.5 | 0.1 | 63.6 | 14.1 | 1.6 | 2.3 | 58.1 |
|  |  |  |  | 4.9 | 0.1 | 0.8 | 2.6 | 2.5 | 0.1 | 65.2 | 14.2 | 1.5 | 2.4 | 57.6 |
|  |  |  |  | 5.2 | 0.1 | 0.8 | 2.4 | 2.5 | 0.1 | 64.9 | 14.3 | 1.5 | 2.3 | 58.4 |
|  |  |  |  | 4.9 | 0.1 | 0.8 | 1.6 | 2.5 | 0.1 | 65.4 | 14.6 | 1.4 | 2.2 | 61.1 |
|  |  |  |  | 5.1 | 0.1 | 0.7 | 2.9 | 2.6 | 0.1 | 63.4 | 14.2 | 2.1 | 2.2 | 58.3 |
|  |  |  |  | 4.7 | 0.1 | 0.8 | 2.0 | 2.6 | 0.1 | 64.8 | 14.5 | 1.8 | 2.3 | 58.8 |
|  |  |  |  | 4.4 | 0.1 | 0.8 | 1.6 | 2.6 | 0.1 | 66.1 | 14.8 | 1.7 | 2.3 | 60.8 |
|  |  |  |  | 4.1 | 0.1 | 0.7 | 1.6 | 2.5 | 0.1 | 66.3 | 14.3 | 1.5 | 2.4 | 60.2 |
|  |  |  |  | 5.1 | 0.1 | 0.7 | 3.0 | 2.5 | 0.1 | 63.4 | 14.0 | 2.0 | 2.2 | 58.0 |
|  |  |  |  | 4.7 | 0.1 | 0.8 | 2.4 | 2.5 | 0.1 | 64.5 | 14.4 | 1.8 | 2.2 | 58.8 |
|  |  |  |  | 4.2 | 0.1 | 0.8 | 1.3 | 2.5 | 0.1 | 65.7 | 14.7 | 1.4 | 2.2 | 62.6 |
|  |  |  |  | 4.9 | 0.1 | 0.8 | 1.5 | 2.5 | 0.1 | 65.3 | 14.5 | 1.3 | 2.3 | 61.2 |
|  |  |  |  | 4.6 | 0.1 | 0.8 | 1.4 | 2.6 | 0.1 | 66.3 | 14.8 | 1.3 | 2.3 | 62.3 |
|  |  |  |  | 4.7 | 0.1 | 0.8 | 3.0 | 2.7 | 0.2 | 63.2 | 14.0 | 2.4 | 2.3 | 57.4 |
|  |  |  |  | 4.8 | 0.1 | 0.7 | 2.8 | 2.6 | 0.2 | 63.9 | 13.9 | 2.0 | 2.4 | 56.9 |
|  |  |  |  | 4.7 | 0.1 | 0.7 | 3.4 | 2.7 | 0.2 | 61.7 | 13.9 | 2.5 | 2.2 | 58.1 |
|  |  |  |  | 4.5 | 0.1 | 0.7 | 3.1 | 2.6 | 0.2 | 59.2 | 13.4 | 2.5 | 2.1 | 58.0 |
|  |  |  |  | 4.6 | 0.1 | 0.7 | 3.1 | 2.6 | 0.2 | 59.8 | 13.6 | 2.4 | 2.1 | 57.9 |
|  |  |  |  | 4.7 | 0.1 | 0.7 | 3.3 | 2.7 | 0.2 | 60.4 | 13.6 | 2.5 | 2.2 | 57.7 |
|  |  |  |  | 4.9 | 0.1 | 0.7 | 3.5 | 2.6 | 0.2 | 60.9 | 13.7 | 2.5 | 2.2 | 58.0 |
|  |  |  |  | 4.7 | 0.1 | 0.7 | 3.1 | 2.6 | 0.2 | 61.1 | 13.8 | 2.5 | 2.2 | 58.0 |
|  |  |  |  | 4.8 | 0.1 | 0.7 | 3.5 | 2.6 | 0.2 | 60.6 | 13.6 | 2.6 | 2.1 | 58.0 |
|  |  |  |  | 4.9 | 0.1 | 0.7 | 3.8 | 2.6 | 0.2 | 60.1 | 13.6 | 2.6 | 2.1 | 58.1 |
|  |  |  |  | 4.8 | 0.1 | 0.7 | 3.8 | 2.6 | 0.2 | 60.5 | 13.6 | 2.6 | 2.1 | 58.0 |
|  |  |  |  | 4.9 | 0.1 | 0.7 | 3.5 | 2.6 | 0.2 | 60.4 | 13.7 | 2.5 | 2.1 | 58.1 |
|  |  |  |  | 4.9 | 0.1 | 0.7 | 3.6 | 2.6 | 0.2 | 60.9 | 13.7 | 2.5 | 2.2 | 58.1 |
|  |  |  |  | 4.7 | 0.1 | 0.7 | 3.5 | 2.6 | 0.2 | 60.7 | 13.7 | 2.5 | 2.1 | 58.2 |
|  |  |  |  | 4.8 | 0.1 | 0.7 | 3.3 | 2.6 | 0.2 | 61.2 | 13.8 | 2.4 | 2.2 | 58.2 |
|  |  |  |  | 4.8 | 0.1 | 0.7 | 3.5 | 2.5 | 0.2 | 60.9 | 13.7 | 2.4 | 2.2 | 58.1 |
|  |  |  |  | 4.8 | 0.1 | 0.7 | 3.8 | 2.6 | 0.2 | 60.5 | 13.6 | 2.5 | 2.1 | 58.2 |
| Zech et al.^27^ | Verkhoyansk Mountains, Siberia | Loess/  Paleosol | < 2 mm | 4.7 | 0.1 | 0.7 | 3.5 | 2.6 | 0.2 | 60.2 | 13.6 | 2.4 | 2.1 | 58.0 |
|  |  |  |  | 4.5 | 0.1 | 0.7 | 2.8 | 2.6 | 0.2 | 60.5 | 13.7 | 2.4 | 2.1 | 58.2 |
|  |  |  |  | 4.6 | 0.1 | 0.7 | 3.0 | 2.6 | 0.2 | 59.1 | 13.4 | 2.4 | 2.1 | 58.2 |
|  |  |  |  | 4.6 | 0.1 | 0.7 | 2.7 | 2.6 | 0.2 | 60.1 | 13.6 | 2.3 | 2.2 | 57.6 |
|  |  |  |  | 5.0 | 0.1 | 0.7 | 3.5 | 2.5 | 0.2 | 59.9 | 13.5 | 2.2 | 2.1 | 58.3 |
|  |  |  |  | 4.9 | 0.1 | 0.7 | 3.2 | 2.6 | 0.2 | 61.6 | 13.8 | 2.2 | 2.2 | 57.6 |
|  |  |  |  | 4.7 | 0.1 | 0.7 | 2.8 | 2.6 | 0.2 | 62.3 | 13.9 | 2.2 | 2.3 | 57.7 |
|  |  |  |  | 4.8 | 0.1 | 0.7 | 2.7 | 2.6 | 0.2 | 62.3 | 13.9 | 2.1 | 2.3 | 57.6 |
|  |  |  |  | 4.6 | 0.1 | 0.7 | 2.5 | 2.6 | 0.2 | 62.4 | 13.9 | 2.2 | 2.3 | 57.4 |
|  |  |  |  | 4.8 | 0.1 | 0.7 | 2.7 | 2.5 | 0.2 | 63.5 | 13.9 | 2.0 | 2.3 | 57.4 |
|  |  |  |  | 4.8 | 0.1 | 0.7 | 2.5 | 2.4 | 0.2 | 62.8 | 13.7 | 1.9 | 2.3 | 57.1 |
|  |  |  |  | 4.6 | 0.1 | 0.8 | 2.4 | 2.6 | 0.2 | 62.5 | 14.0 | 2.1 | 2.3 | 57.4 |
|  |  |  |  | 4.6 | 0.1 | 0.7 | 2.1 | 2.4 | 0.2 | 63.7 | 13.8 | 1.8 | 2.4 | 57.1 |
|  |  |  |  | 4.7 | 0.1 | 0.7 | 1.8 | 2.7 | 0.2 | 66.8 | 14.2 | 1.8 | 2.4 | 58.5 |
|  |  |  |  | 4.6 | 0.1 | 0.7 | 1.5 | 2.7 | 0.2 | 67.6 | 14.1 | 1.7 | 2.5 | 59.2 |
|  |  |  |  | 4.5 | 0.1 | 0.7 | 1.8 | 2.6 | 0.2 | 67.2 | 13.8 | 1.8 | 2.4 | 57.7 |
|  |  |  |  | 4.5 | 0.1 | 0.7 | 2.1 | 2.6 | 0.2 | 66.3 | 13.9 | 1.9 | 2.4 | 56.7 |
|  |  |  |  | 4.9 | 0.1 | 0.7 | 2.1 | 2.7 | 0.2 | 65.6 | 14.1 | 2.0 | 2.4 | 57.1 |
|  |  |  |  | 4.9 | 0.1 | 0.7 | 2.0 | 2.7 | 0.2 | 65.6 | 14.0 | 1.8 | 2.4 | 57.2 |
|  |  |  |  | 4.5 | 0.1 | 0.7 | 2.0 | 2.7 | 0.2 | 65.4 | 14.0 | 1.9 | 2.4 | 57.3 |
|  |  |  |  | 4.4 | 0.1 | 0.7 | 1.2 | 2.4 | 0.1 | 68.6 | 13.8 | 1.3 | 2.5 | 60.7 |
|  |  |  |  | 4.1 | 0.1 | 0.7 | 1.2 | 2.3 | 0.1 | 70.1 | 13.0 | 1.2 | 2.5 | 59.9 |
|  |  |  |  | 3.9 | 0.1 | 0.6 | 1.0 | 2.3 | 0.1 | 71.5 | 12.9 | 1.1 | 2.4 | 60.8 |
|  |  |  |  | 3.6 | 0.1 | 0.6 | 1.0 | 2.1 | 0.1 | 73.2 | 12.1 | 1.0 | 2.4 | 59.7 |
|  |  |  |  | 3.8 | 0.0 | 0.6 | 1.0 | 2.2 | 0.1 | 72.5 | 12.8 | 1.1 | 2.4 | 61.1 |
|  |  |  |  | 5.2 | 0.1 | 0.7 | 4.9 | 2.7 | 0.2 | 60.3 | 14.1 | 2.2 | 2.3 | 56.9 |
|  |  |  |  | 5.0 | 0.1 | 0.7 | 5.1 | 2.7 | 0.2 | 59.5 | 13.8 | 2.2 | 2.3 | 56.9 |
|  |  |  |  | 3.4 | 0.1 | 0.6 | 3.5 | 2.3 | 0.2 | 69.2 | 11.6 | 1.4 | 2.6 | 51.5 |
|  |  |  |  | 3.0 | 0.1 | 0.5 | 2.4 | 2.2 | 0.1 | 73.8 | 10.8 | 1.1 | 2.5 | 50.0 |
|  |  |  |  | 2.7 | 0.0 | 0.6 | 1.3 | 2.2 | 0.1 | 75.6 | 11.4 | 0.8 | 2.8 | 54.7 |
|  |  |  |  | 2.3 | 0.0 | 0.6 | 1.1 | 2.1 | 0.1 | 72.4 | 11.9 | 0.8 | 2.9 | 56.9 |
|  |  |  |  | 2.7 | 0.1 | 0.6 | 1.4 | 2.3 | 0.1 | 72.8 | 12.0 | 0.9 | 2.8 | 55.8 |
| Hoffmann  et al.^28^ | Isua Supracrustal Belt, Southwestern Greenland | Amphibolite | Bulk | 5.1 | 0.0 | 0.3 | 2.2 | 1.6 | 0.0 | 72.8 | 11.9 | 1.4 | 2.6 | 54.7 |
|  |  |  |  | 10.4 | 0.1 | 0.4 | 1.1 | 1.7 | 0.1 | 62.1 | 15.1 | 2.6 | 3.7 | 60.5 |
|  |  |  |  | 4.8 | 0.0 | 0.5 | 2.6 | 1.9 | 0.1 | 66.6 | 15.5 | 1.9 | 4.1 | 53.6 |
|  |  |  |  | 11.4 | 0.1 | 0.6 | 1.5 | 0.2 | 0.1 | 56.1 | 17.7 | 3.0 | 5.7 | 59.1 |
| Hoffmann  et al.^29^ |  |  |  | 11.3 | 0.2 | 0.8 | 9.2 | 0.1 | 0.0 | 51.9 | 13.6 | 9.6 | 2.0 | 66.5 |
|  |  |  |  | 17.5 | 0.3 | 1.0 | 9.8 | 0.2 | 0.1 | 45.9 | 12.8 | 9.4 | 1.4 | 72.2 |
|  |  |  |  | 13.2 | 0.2 | 0.9 | 10.8 | 0.1 | 0.1 | 48.4 | 14.6 | 7.3 | 1.9 | 69.2 |
|  |  |  |  | 11.8 | 0.2 | 0.8 | 8.9 | 0.3 | 0.1 | 53.9 | 14.0 | 5.1 | 3.7 | 52.9 |
|  |  |  |  | 11.9 | 0.2 | 0.7 | 9.4 | 0.1 | 0.0 | 53.9 | 8.6 | 12.6 | 1.5 | 62.7 |
|  |  |  |  | 12.4 | 0.2 | 0.8 | 10.5 | 0.1 | 0.0 | 51.4 | 9.3 | 12.6 | 1.5 | 65.5 |
| Hoffmann  et al.^29^ | Isua Supracrustal Belt, Southwestern Greenland | Amphibolite | Bulk | 12.2 | 0.2 | 0.7 | 10.2 | 0.1 | 0.0 | 53.4 | 8.2 | 12.6 | 1.2 | 66.3 |
|  |  |  |  | 10.7 | 0.2 | 0.7 | 8.9 | 0.1 | 0.0 | 52.0 | 13.6 | 10.2 | 1.7 | 70.3 |
|  |  |  |  | 13.1 | 0.2 | 0.5 | 8.7 | 0.4 | 0.0 | 49.0 | 6.7 | 18.4 | 0.6 | 72.4 |
|  |  |  |  | 14.3 | 0.2 | 0.6 | 9.2 | 0.1 | 0.0 | 47.0 | 7.2 | 17.4 | 0.7 | 75.0 |
| Taylor & McLennan^30^ | UCC | | | 4.5 | 0.1 | 0.5 | 4.2 | 3.4 | 0.2 | 65.9 | 15.2 | 2.2 | 3.9 | 47.9 |


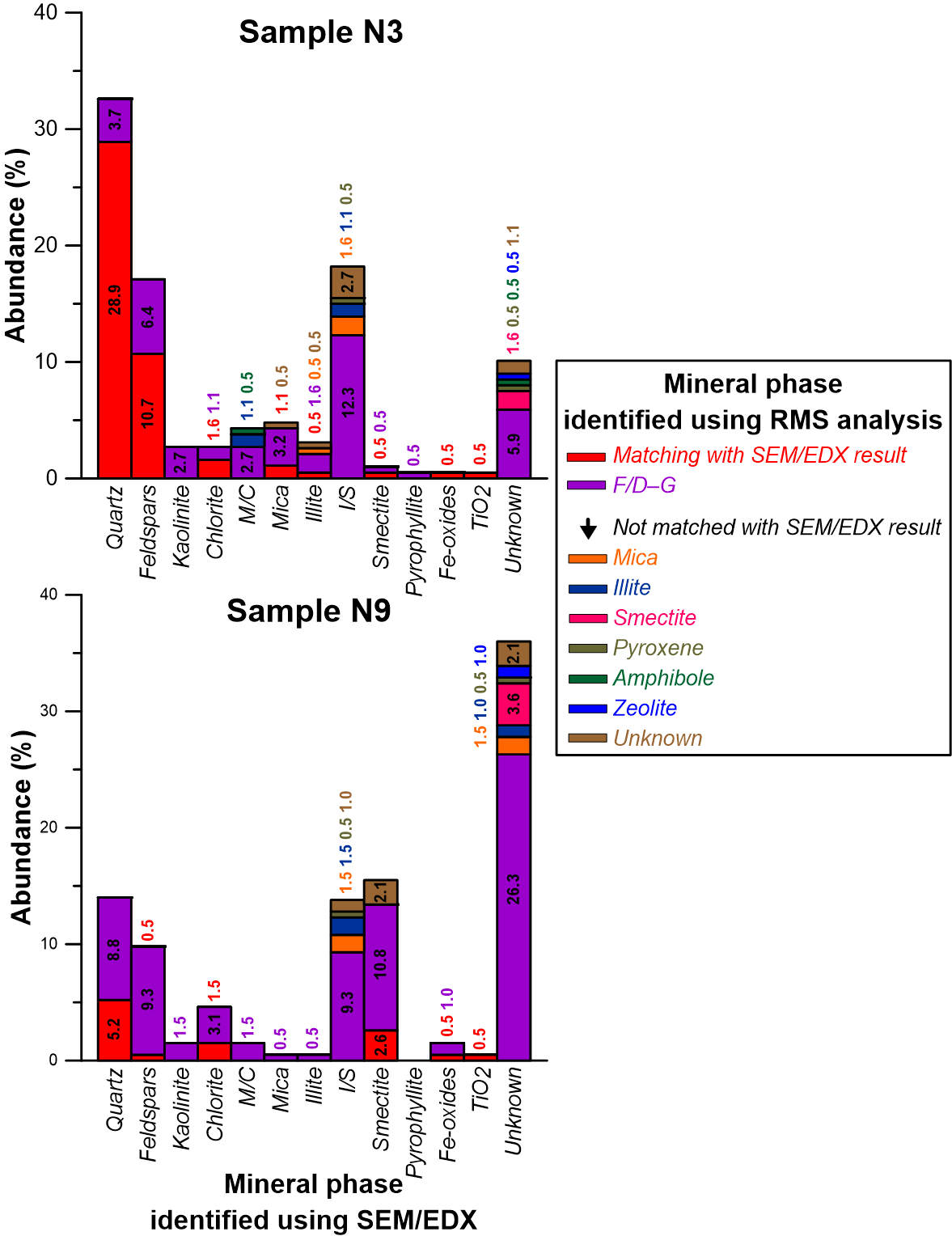


**Figure S1**. Relative abundance (%) of minerals identified by RMS with respect to the mineral phases determined from the same individual particles by SEM/EDX analysis for samples nos. N3 and N9 (also see **Table 1**). Feldspars involve plagioclase (Na– and Ca–feldspar), K–feldspar, and feldspar mixtures. M/C and I/S represent mica/chlorite and illite/smectite mixed layer clays, respectively. Unknown indicates particles that could not be identified using SEM/EDX and RMS.


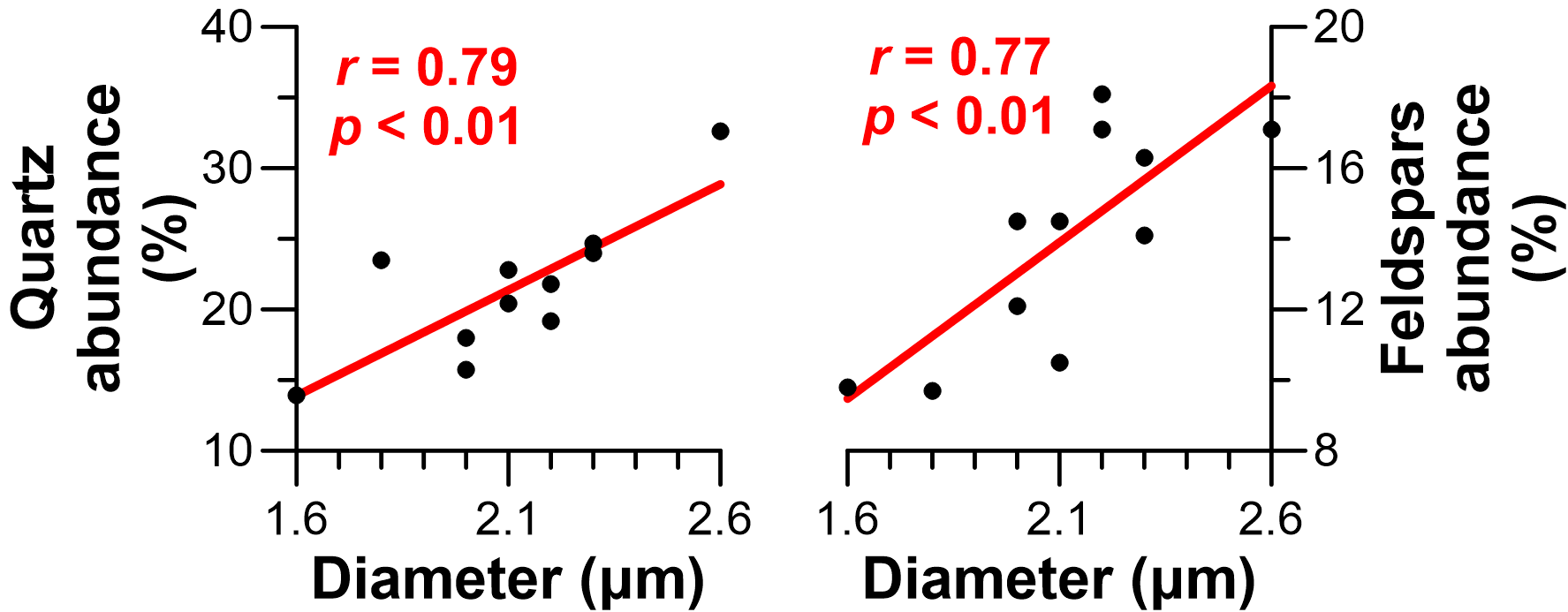


**Figure S2.** The least squares regression lines are shown for abundances in quartz (left) (Pearson’s correlation coefficient of 0.79 at *p* < 0.01) and feldspars (right) (Pearson’s correlation coefficient of 0.77 at *p* < 0.01) with respect to mean particle diameter.


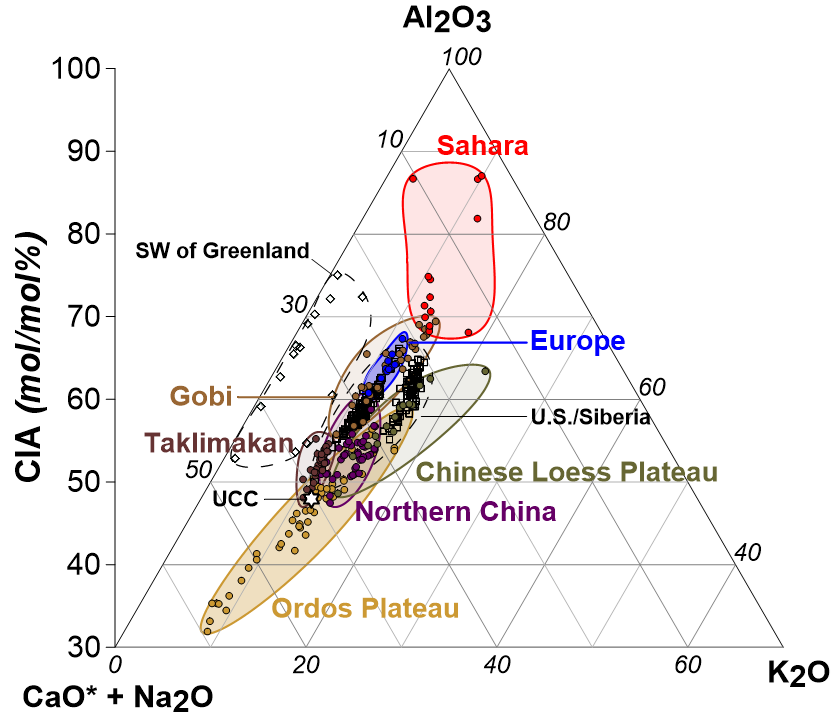


**Figure S3**. A-CN-K ternary diagram in molecular proportions with CIA values of PSAs from published literature: the western territories of North Africa (a representative of Saharan dust; red circle, < 63 μm)^18–24^; European loess deposits (blue circle, < 2 μm)^25^; the Gobi Desert (brown circle, < 20 μm)^15^; the Chinese Loess Plateau (olive circle, < 2 μm)^16^; the Taklimakan Desert (dark brown circle, < 63 μm)^13–14^; northern China (purple circle, < 63 μm)^17^; the Ordos Plateau (gold circle, < 2 μm)^16^; North American and Siberian loess deposits (square, bulk)^26,27^; and southern West Greenland (diamond, bulk)^28,29^ (see **Supplementary** **Table S1**). Also shown is CIA value of upper continental crust (UCC, star)^30^.


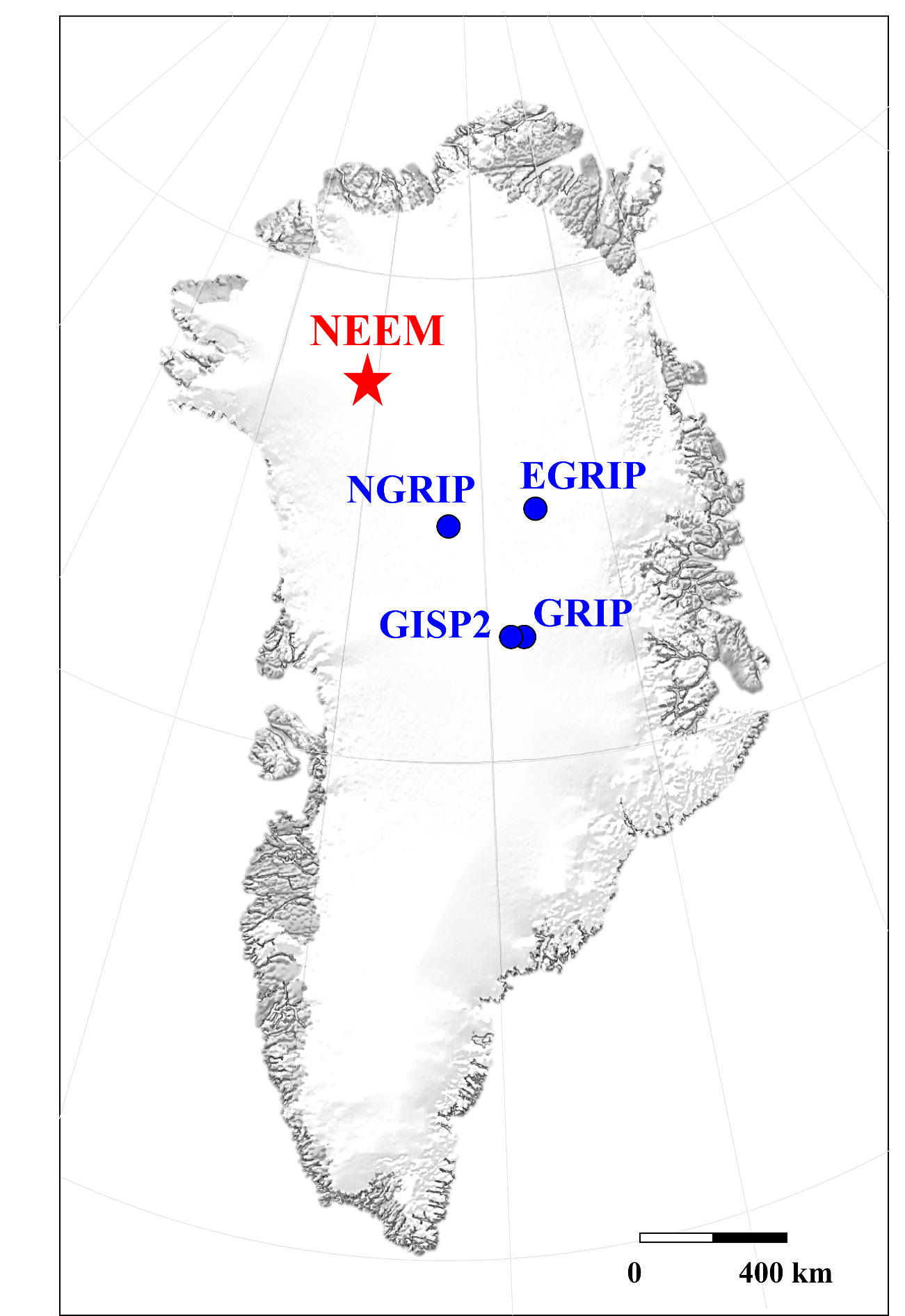


**Figure S4**. Map of Greenland, showing the locations of the deep ice core drilling sites that are discussed in the text: NEEM (North Greenland Eemian Ice Drilling), NGRIP (North Greenland Ice Core Project), EGRIP (East Greenland Ice Core Project), GRIP (Greenland Ice Core Project), and GISP2 (Greenland Ice Sheet Project 2).


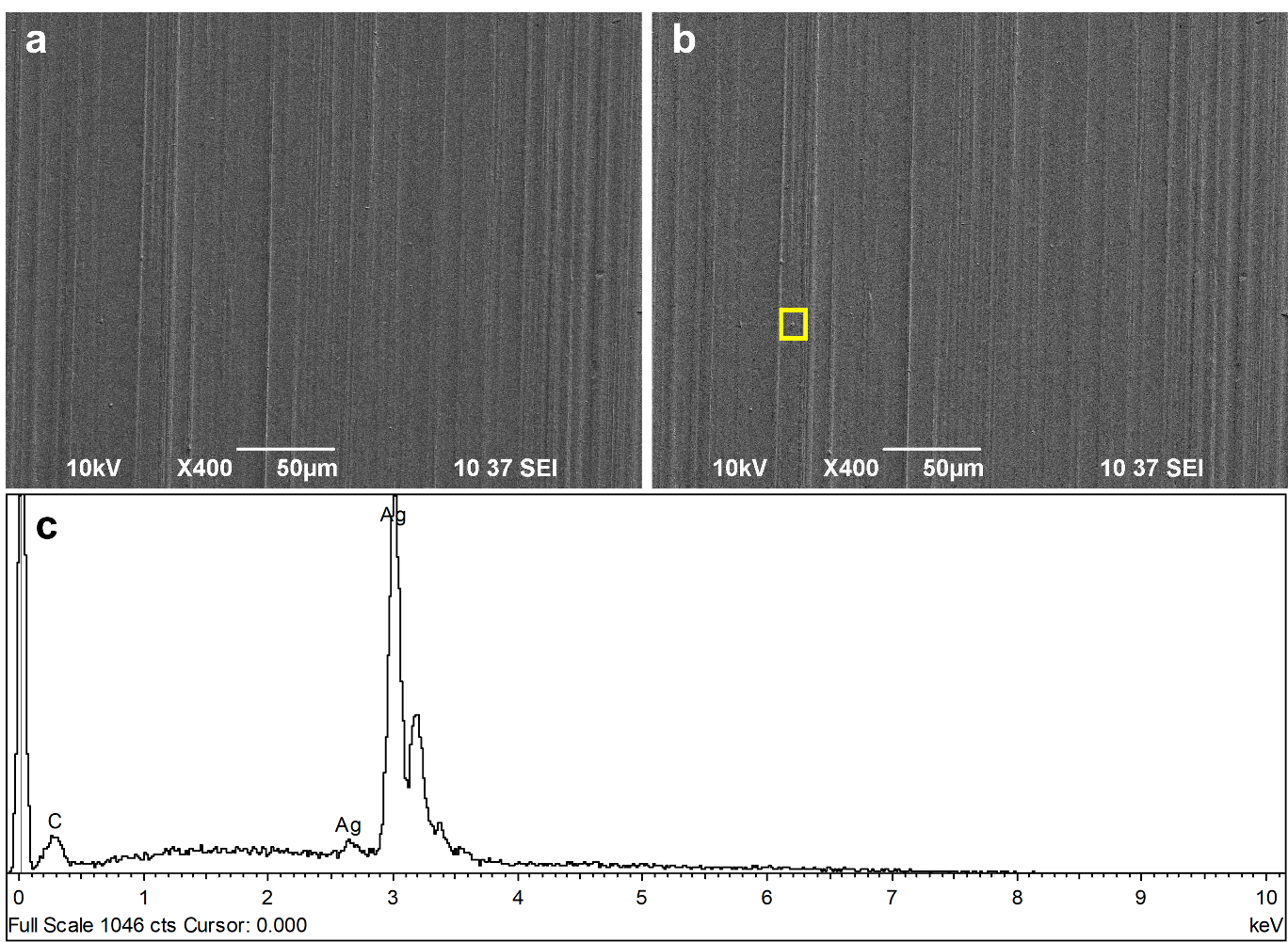


**Figure S5**. Secondary electron images (SEIs) of SEM for an Ag foil substrate (a) before and (b) after loading ultrapure water droplets. The small-sized particles observed on the pure Ag foil in (a) were Ag-bearing particles existing on the Ag foil surface, which can be easily identified using SEM/EDX (c). A particle in the yellow box in (b) represents a particle introduced after loading ultrapure water droplet.

**Figure S6**. Typical X-ray spectra of feldspars, quartz, kaolinite, pyrophyllite, Fe–oxides, and TiO_2_ observed in Greenland NEEM ice core samples.

**
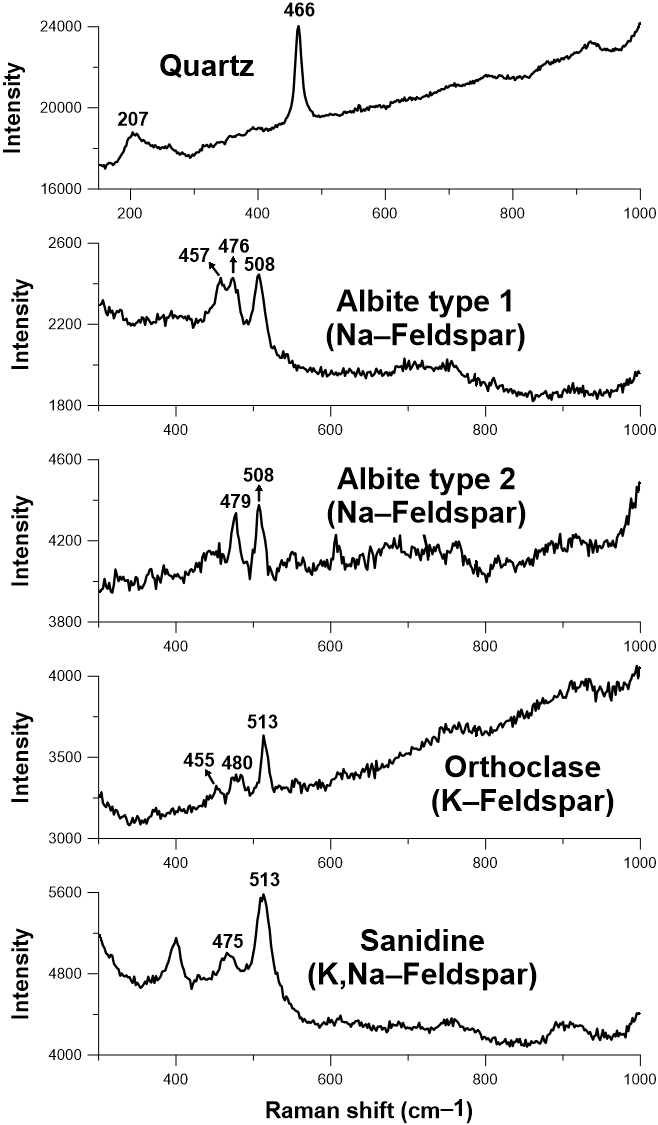
**

**Figure S7**. Typical Raman spectra of different minerals observed in Greenland NEEM ice core samples.


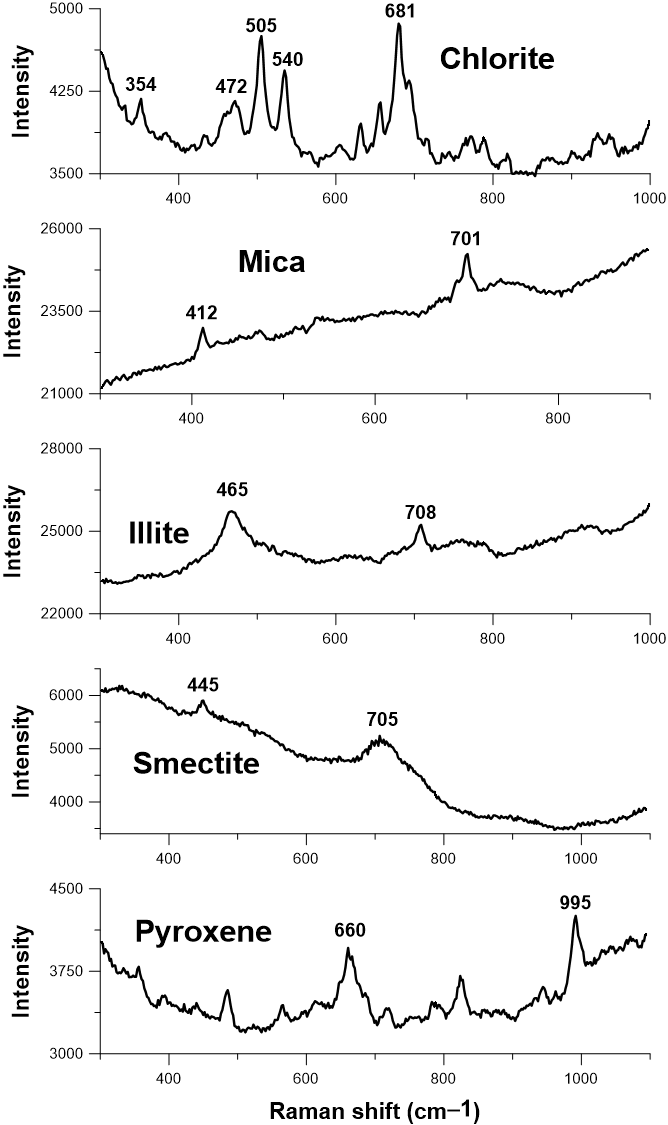


**Figure S7**. (continued).


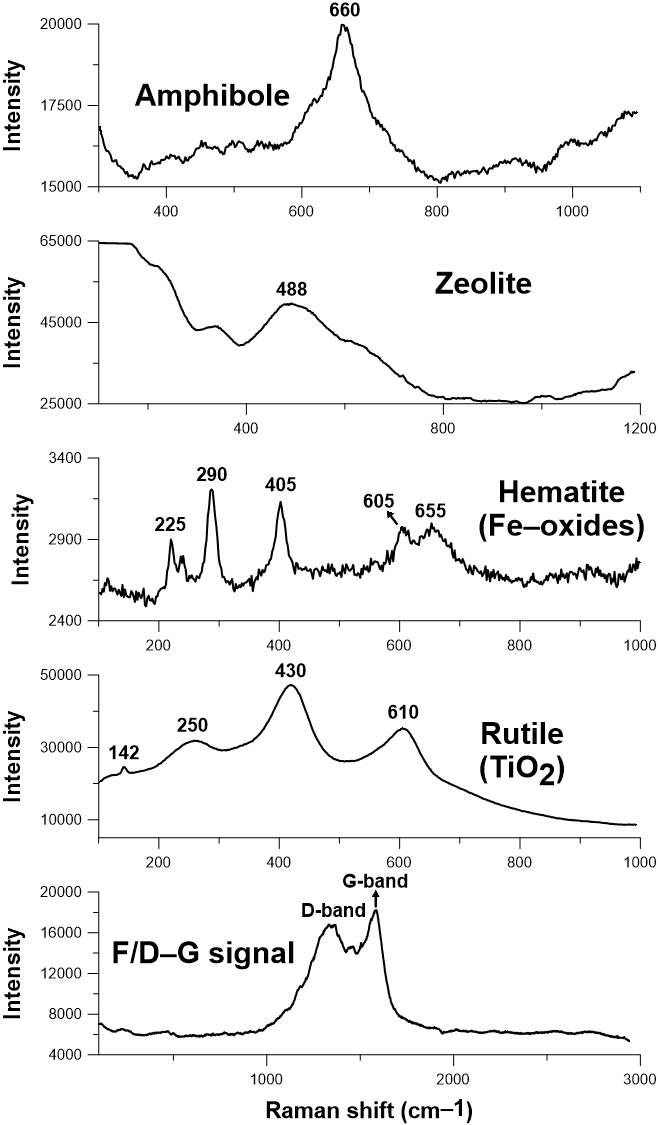


**Figure S7**. (continued).


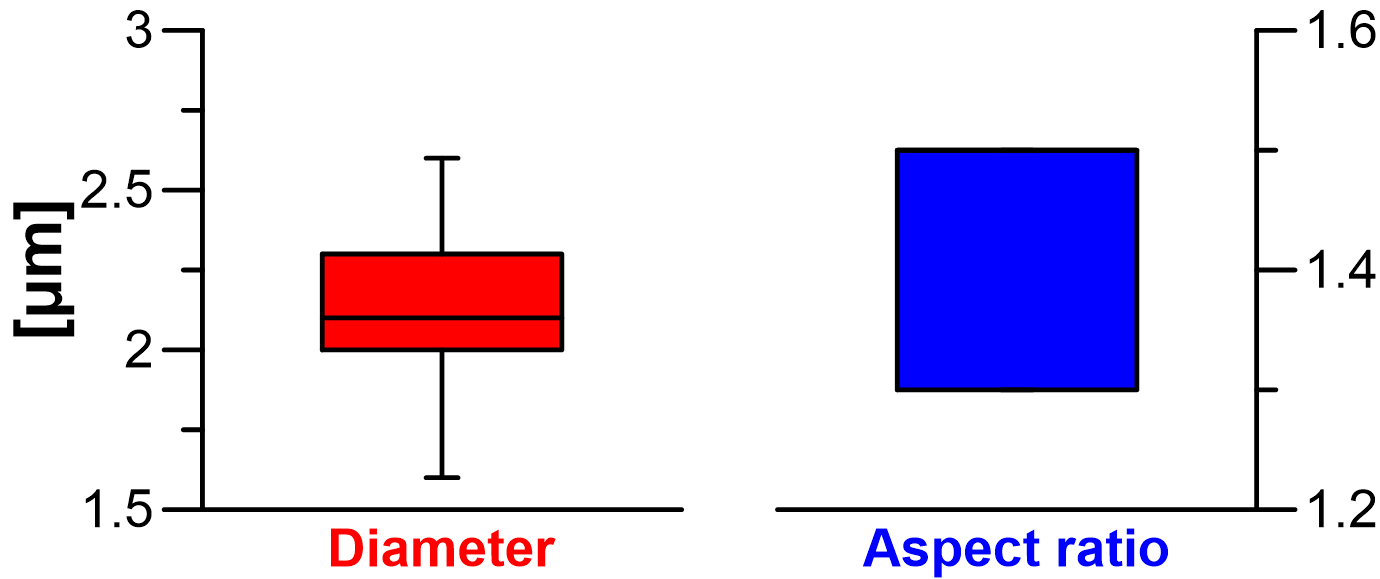


**Figure S8**. Box plots of mean particle diameters (red) and sapect ratios (blue) determined in individual samples. The boundaries of the box are Tukey’s hinges. The median is identified by the line inside the box and the length of the box represents the interquartile range (IQR) computed from Tukey’s hinge. Values more than three IQR’s from the end of a box are considered outliers.


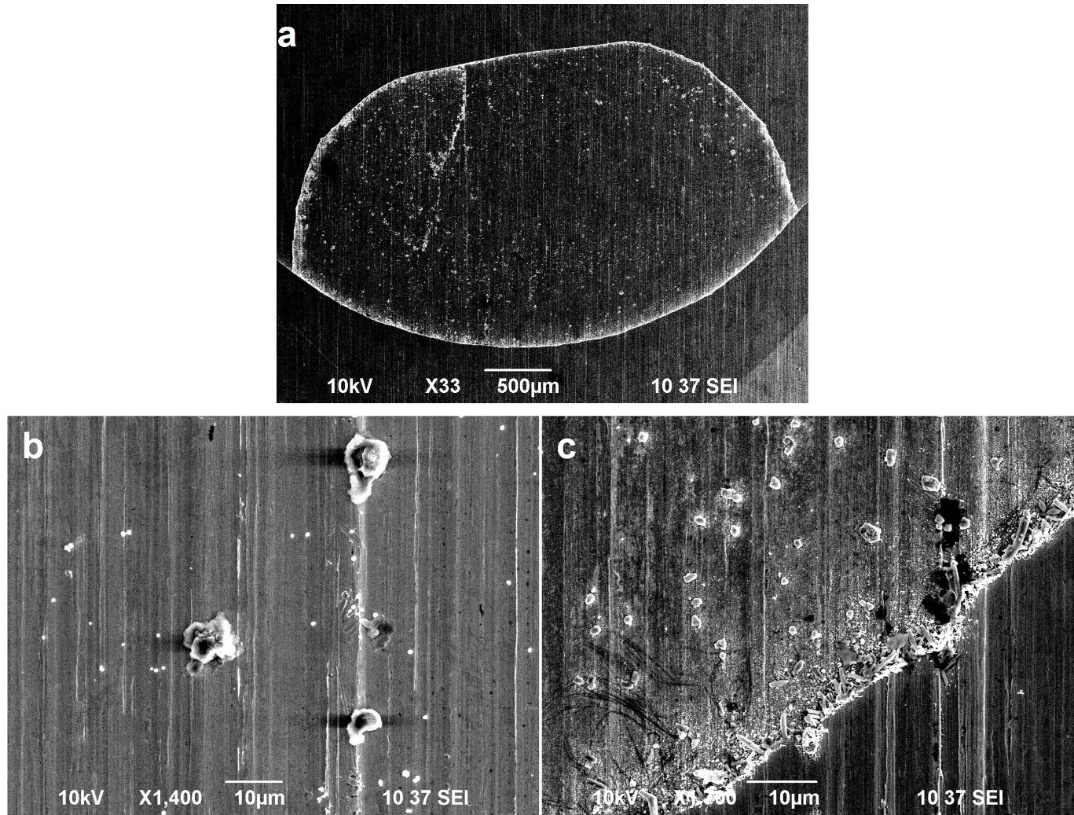


**Figure S9**. Secondary electron images (SEIs) of evaporated kaolinite (overall (a), central (b), and side (c) sections) after a droplet of standard reference material (SRM) containing kaolinite was loaded onto an Ag foil using a microdrop deposition method. We observed that these SEIs were identical for the two methods that evaporated SRM droplets at room temperature and 100˚C in an oven, respectively.


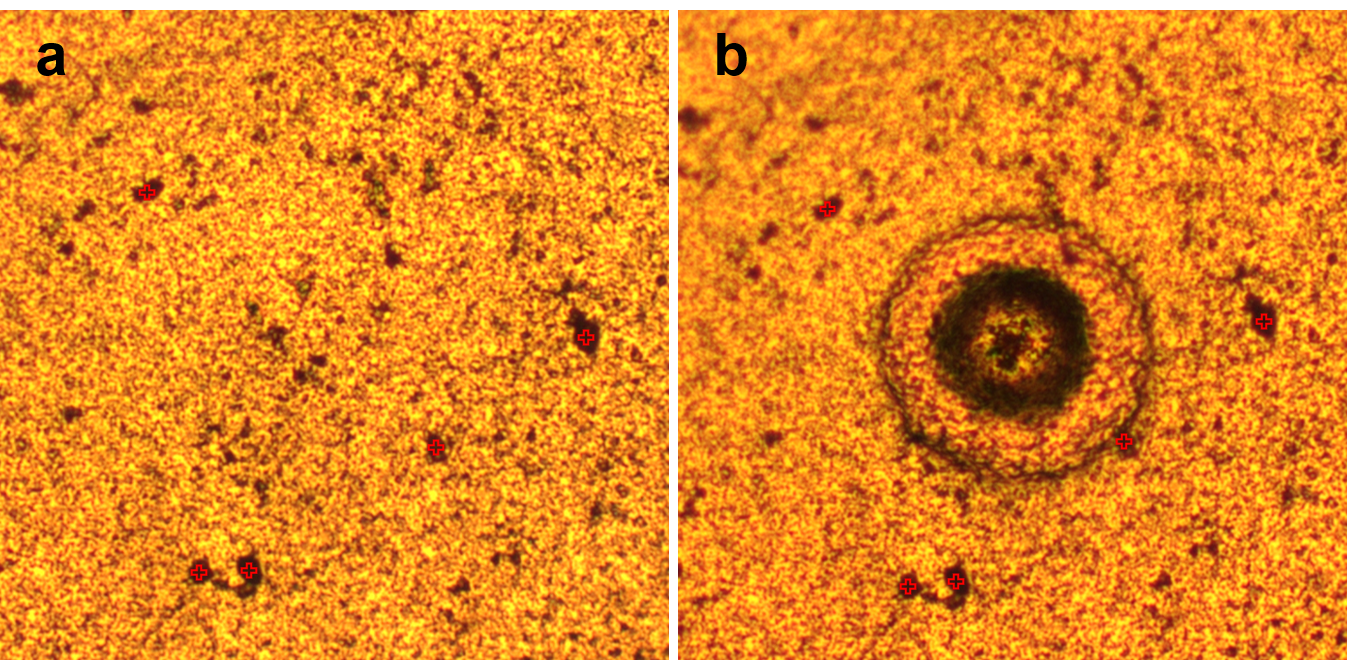


**Figure S10**. Optical field images of particles in Greenland NEEN ice appearing as black stain-like features (e.g., red crosses) loaded onto an Au-coated polycarbonate filter before (a) and after (b) Raman measurement using a laser beam of ~11 mW. The dual ring-like signature in (b) represents a deformation feature of the Au-coated polycarbonate filter after Raman me.

**References**

1. Murr, L. E., Esquivel, E. V., Bang, J. J., de la Rosa, G. & Gardea-Torresdey, J. L. Chemistry and nanoparticulate compositions of a 10,000 year-old ice core melt water. *Water Res.* **38**, 4282-4296 (2004).

2. Paleari, C. I. *et al.* Aeolian dust provenance in central East Antarctica during the Holocene: Environmental constraints from single‐grain Raman spectroscopy. *Geophys. Res. Lett.* **46**, 9968-9979 (2019).

3. Macis, S. *et al.* Microdrop deposition technique: Preparation and characterization of diluted suspended particulate samples. *Condens. Matter* **3**, 21; 10.3390/condmat3030021 (2018).

4. Innocenzi, P., Malfatti, L., Piccinini, M., Grosso, D. & Marcelli, A. Stain effects studied by time-resolved infrared imaging. *Anal. Chem*. **81**, 551-556 (2009).

5. Steffensen, J. P. The size distribution of microparticles from selected segments of the Greenland Ice Core Project ice core representing different climatic periods. *J. Geophys. Res. Oceans* **102**, 26755-26763 (1997).

6. Ruth, U., Wagenbach, D., Steffensen, J. P. & Bigler, M. Continuous record of microparticle concentration and size distribution in the central Greenland NGRIP ice core during the last glacial period. *J. Geophys. Res. Atmos.* **108**, 4098; 10.1029/2002jd002376 (2003).

7. Briat, M., Royer, A., Petit, J. R. & Lorius, C. Late glacial input of eolian continental dust in the Dome C ice core: Additional evidence from individual microparticle analysis. *Ann. Glaciol.* **3**, 27-31 (1982).

8: Gaudichet, A. *et al.* Comments of the origin of dust in East Antarctica for present and ice age conditions. *J. Atmos. Chem.* **14**, 129-142 (1992).

9. Wu, G., Zhang, X., Zhang, C. & Xu, T. Mineralogical and morphological properties of individual dust particles in ice cores from the Tibetan Plateau. *J. Glaciol.* **62**, 46-53 (2016).

10. Haynes, W. M. *CRC Handbook of Chemistry and Physics* (CRC press, 2014).

11. Khan, M. S., Hwang, H., Kim, H. & Ro, C. U. Molecular mass concentrations for a powdered SRM sample using a quantitative single particle analysis. *Anal. Chim. Acta* 619, 14-19 (2008).

12. Malek, M. A. *et al.* Single particle mineralogy of microparticles from Himalayan ice-cores using SEM/EDX and ATR-FTIR imaging techniques for identification of volcanic ash signatures. *Chem. Geol.* **504**, 205-215 (2019).

13. Jiang, Q. & Yang, X. Sedimentological and geochemical composition of aeolian sediments in the Taklamakan Desert: Implications for provenance and sediment supply mechanisms. *J. Geophys.* *Res. Earth Surf.* **124**, 1217-1237 (2019).

14. Yang, X., Zhu, B. & White, P. D. Provenance of aeolian sediment in the Taklamakan Desert of western China, inferred from REE and major-elemental data. *Quat. Int.* **175**, 71-85 (2007).

15. Jeong, G. Y. Mineralogy and geochemistry of Asian dust: dependence on migration path, fractionation, and reactions with polluted air. *Atmos. Chem. Phys.* **20**, 7411-7428 (2020).

16. Pang, H. *et al.* Mineralogy and geochemistry of modern Yellow River sediments: Implications for weathering and provenance. *Chem. Geol.* **488**, 76-86 (2018).

17. Xie, Y. & Chi, Y. Geochemical investigation of dry- and wet-deposited dust during the same dust-storm event in Harbin, China: Constraint on provenance and implications for formation of aeolian loess. *J. Asian Earth Sci.* **120**, 43-61 (2016).

18. Bücher, A. & Lucas, C. Sédimentation éolienne intercontinentale, poussières sahariennes et géologie. *Bull. Cent. Rech. Explor. Prod.* **8**, 151-165 (1984).

19. Bücher, A. *Fallout of Saharan dust in the northern Mediterranean region* (Kluwer Academic Publishers, 1989).

20. Orange, D., Gac, J. Y. & Diallo, M. I. Geochemical assessment of atmospheric deposition including Harmattan dust in continental West Africa*.* In *Tracers in Hydrology: Proc. Yokohama Symposium* (ed. Peters, N. E., Hoehn, E., Leibundgut, C., Tase, N. & Walling, D. E.) 303-312 (IAHS, 1993).

21. Herrmann, L., Jahn, R. & Stahr, K. *Identiﬁcation and quantiﬁcation of dust additions in peri-Saharan soils* (Kluwer Academic Publishers, 1996).

22. Criado, C. & Dorta, P. An unusual ‘blood rain’ over the Canary Islands (Spain). The storm of January 1999. *J. Arid Environ.* **55**, 765-783 (2003).

23. Linke, C. *et al.* Optical properties and mineralogical composition of different Saharan mineral dust samples: a laboratory study. *Atmos. Chem. Phys.* **6**, 3315-3323 (2006).

24. Castillo, S. *et al.* Trace element variation in size-fractionated African desert dusts. *J. Arid Environ.* **72**, 1034-1045 (2008).

25. Fu, Y. *et al.* Clay mineralogy of the Stari Slankamen (Serbia) loess-paleosol sequence during the last glacial cycle — Implications for dust provenance and interglacial climate. *Quat. Sci. Rev.* **263**, 106990; 10.1016/j.quascirev.2021.106990 (2021).

26. Muhs, D. R. *et al.* Origin and paleoclimatic significance of late Quaternary loess in Nebraska: Evidence from stratigraphy, chronology, sedimentology, and geochemistry. *Geol. Soc. Am. Bull.* **120**, 1378-1407 (2008).

27. Zech, M. *et al.* Characterisation and palaeoclimate of a loess-like permafrost palaeosol sequence in NE Siberia. *Geoderma* **143**, 281-295 (2008).

28. Hoffmann, J. E. *et al.* Highly depleted Hadean mantle reservoirs in the sources of early Archean arc-like rocks, Isua supracrustal belt, southern West Greenland. *Geochim. Cosmochim. Acta* **74**, 7236-7260 (2010).

29. Hoffmann, J. E., Münker, C., Polat, A., Rosing, M. T. & Schulz, T. The origin of decoupled Hf–Nd isotope compositions in Eoarchean rocks from southern West Greenland. *Geochim. Cosmochim. Acta* **75**, 6610-6628 (2011).

30. Taylor, S. R. & McLennan, S. M. *The Continental Crust: Its Composition and Evolution* (Blackwell Science Publishing, 1985).
